# Supplementary figures and images for: Myofibrillar protein accumulation but reduced protein synthesis in PDCD4-depleted myotubes
Source: PLoS One. 2026 Mar 19;21(3):e0345305. doi: 10.1371/journal.pone.0345305 (PMC13001914; doi:10.1371/journal.pone.0345305)

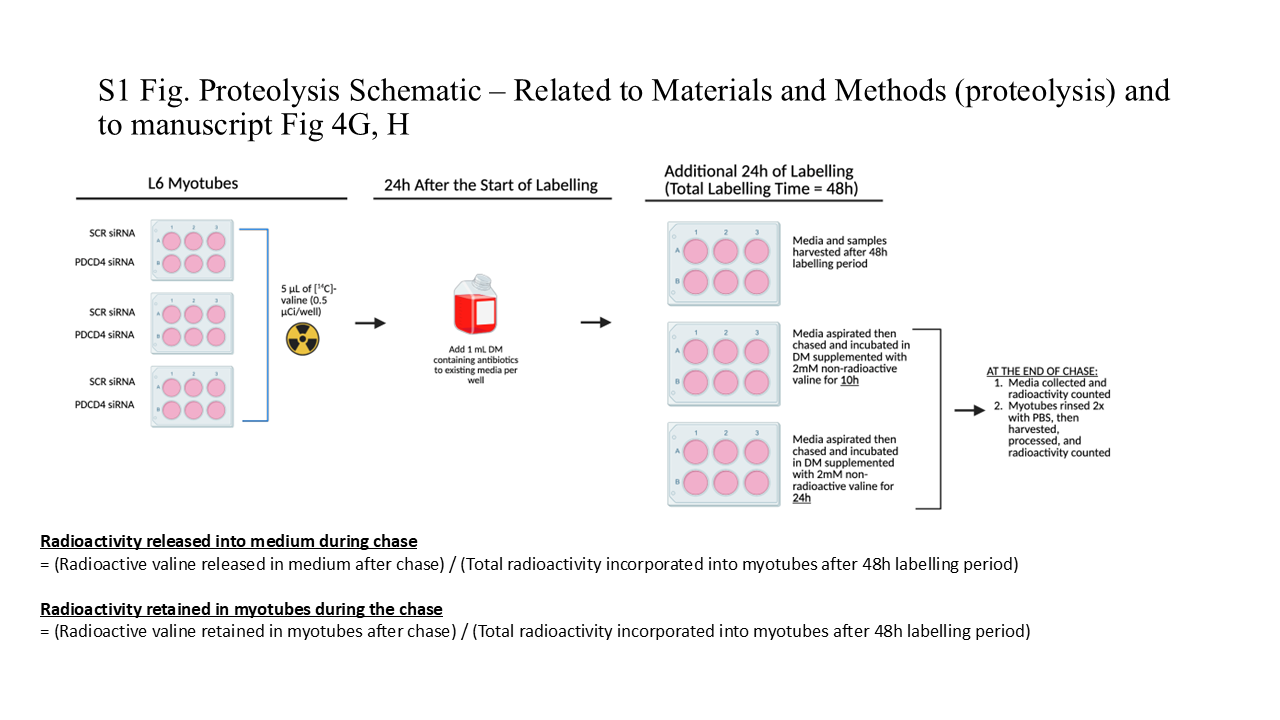

Supplement: S1 Fig — After adding siRNA transfection mix, myotubes were labeled with 0.5uCi/well of 14C-valine. Twenty-four h later, I mL of DM was added and incubation continued for another 24 h. At the end of 48 h of labeling with 14C-valine, aliquots of incubation medium were taken from some batches of wells, along with harvesting of some myotubes. Protein-associated radioactivity in the harvested cells was measured and designated protein-associated radioactivity after the 14C-valine labeling period. The other batches of myotubes were rinsed in PBS and then incubated in the ‘chase’ medium (DM supplemented with 2 mM non-radioactive valine) for 10 or 24 h. Aliquots of the incubation media and as well as batches of myotubes were harvested at the end of the 10 or 24 h chase period. Proteolysis was calculated either as the fraction of radioactivity released into the medium during the chase period relative to protein-associated radioactivity at the end of the labeling period (which would be positively proportional to the rate of proteolysis), or the amount of radioactivity retained in myotubes during chase as a fraction of protein-associated radioactivity at the end of the labeling period (negatively proportional to the rate of proteolysis). (TIF) [file pone.0345305.s001.TIF]

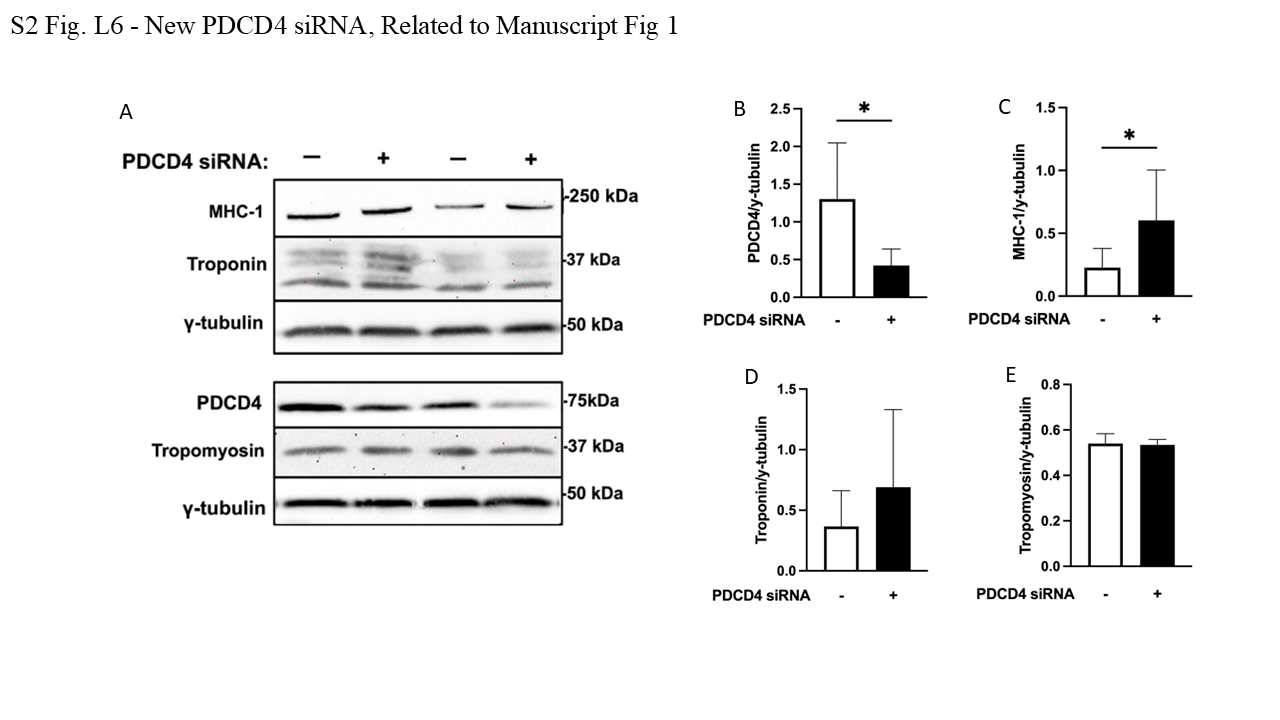

Supplement: S2 Fig — L6 myotubes were transfected with control or siRNA oligonucleotides that are distinct from those used in manuscript Fig 1. Transfection was as described in Fig 1. Western blotting analyses were used to measure the abundance of PDCD4 (A, B), MHC-1 (A, C), troponin (A, D) and tropomyosin (A, E). Data in B-E are presented as mean ± SEM; n = 3 independent experiments with 3 technical replicates per experiment. * p < 0.05. (TIF) [file pone.0345305.s002.TIF]

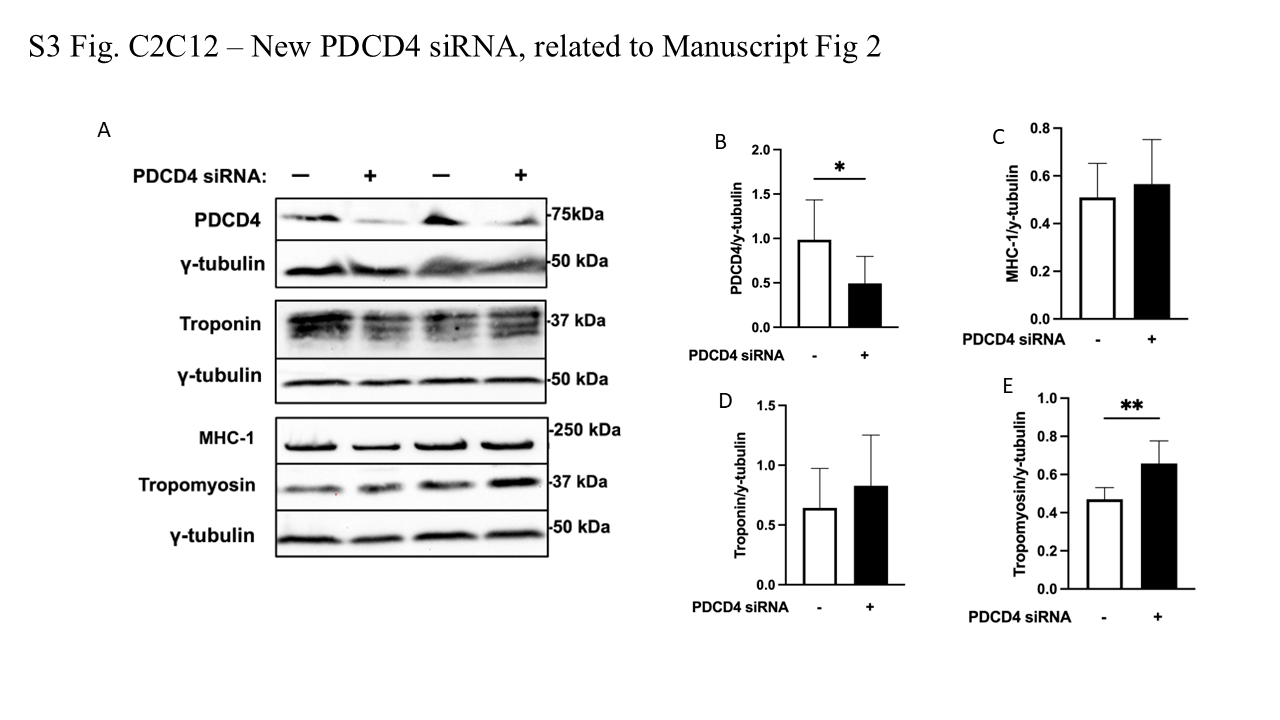

Supplement: S3 Fig — C2C12 myotubes were transfected with control or siRNA oligonucleotides that are distinct from those used in manuscript Fig 2. Transfection was as described in Fig 2. Western blotting analyses were used to measure the abundance of PDCD4 (A, B), MHC-1 (A, C), troponin (A, D) and tropomyosin (A, E). Data in B-E are presented as mean ± SEM; n = 3 independent experiments with at 3 technical replicates per experiment. * p < 0.05; ** p < 0.01. (TIF) [file pone.0345305.s003.TIF]
