# Supplementary material for: Myofibrillar protein accumulation but reduced protein synthesis in PDCD4-depleted myotubes
Source: PLoS One. 2026 Mar 19;21(3):e0345305. doi: 10.1371/journal.pone.0345305 (PMC13001914; doi:10.1371/journal.pone.0345305)
Supplement: S1 File — (PDF) [file pone.0345305.s004.pdf]

Replicates #(n	1A	
	SCR	PDCD4 KO
1	0.805357	0
2	0.800355	0.023943
3	0.442743	0
4	1.221782	0

1C	
SCR	PDCD4 KO
72.20377	87.13044
63.87627	97.5
58.05304	80.20088

1E	
SCR	PDCD4 KO
0.240681	1.543785
0.485998	1.137367
0.746132	1.193293

2B	
SCR	PDCD4 KO
0.851827	0.132147
1.637292	0.238109
1.231279	0.253449

2C	
SCR	PDCD4 KO
0.371651	0.666123
0.476814	0.796194
0.441633	0.760761

2D	
SCR	PDCD4 KO
1.046987	1.237025
0.730079	1.258745
0.780644	1.111394

3B	
SCR	PDCD4 KO
0.686822	1.413003
0.625215	0.936735
0.495375	1.010948

3C	
SCR	PDCD4 KO
0.076308	0.311742
0.093905	0.334859
0.198807	0.249809

3D	
SCR	PDCD4 KO
0.315421	0.316133
0.228007	0.282348
0.298929	0.243813

4B	
SCR	PDCD4 KO
0.348481897	0.533920621
0.175448888	0.960176529
0.28106305	1.296991498

4C	
SCR	PDCD4 KO
0.231538	0.124222
0.253478	0.16865
0.227271	0.101096

4D	
SCR	PDCD4 KO
0.242723	0.261495
0.394441	0.357783
0.374328	0.405433

5A	
SCR	PDCD4 KO
1.04984	1.11047
0.968093	1.5493
0.99342	1.0464
0.8734	0.841476

5B	
SCR	PDCD4 KO
399252	547601
510039	678068
395091	721427
288489	514192

5C	
SCR	PDCD4 KO
2.629517197	2.02788161
1.89807642	2.28487408
2.51440807	1.4504586
3.02749845	1.63650154

Fig 6A

VALINE		LEUCINE		ISOLEUCINE		TOTAL BCAAs	
SCR	PDCD4 KO	SCR	PDCD4 KO	SCR	PDCD4 KO	SCR	PDCD4 KO
0.45857	0.01023	0.41327	0.09370	0.38509	0.03557	0.458565	0.010225
0.20022	0.02032	0.17874	0.07563	0.29591	0.03762	0.200224	0.020318
0.21026	0.02076	0.19566	0.12425	0.44735	0.09121	0.210258	0.020758
0.16781	0.03681	0.14665	0.11655	0.27814	0.20841	0.167814	0.036810
0.15935	0.03146	0.17654	0.11517	0.29103	0.07532	0.159353	0.031464
0.16932	0.03688	0.20473	0.12822	0.20262	0.09038	0.169322	0.036880
0.17735	0.02635	0.17388	0.15671	0.28327	0.09946	0.177353	0.026353
0.17371	0.06650	0.18568	0.16973	0.28308	0.10501	0.173706	0.066495
0.19366	0.04559	0.16644	0.02855	0.38230	0.11109	0.193655	0.045588
						0.413273	0.093703
						0.178742	0.075634
						0.195659	0.124246

0.146649	0.116547
0.176536	0.115175
0.204728	0.128223
0.173878	0.156712
0.185683	0.169733
0.166437	0.028555
0.385095	0.035572
0.295914	0.037618
0.447347	0.091206
0.278140	0.208415
0.291025	0.075322
0.202622	0.090381
0.283270	0.099465
0.283084	0.105006
0.382295	0.111089

7B		
SCR	PDCD4 KO	PDCD4 KO
NO AKT INHIB	NO AKT INHIB	YES AKT INHIB
1.362437	2.543179	0.729623
1.115815	1.755888	0.562616
1.665022	1.807603	0.407112

7C		
SCR	PDCD4 KO	PDCD4 KO
NO AKT INHIB	NO AKT INHIB	YES AKT INHIB
1.196324382	0.118078	0.409164
1.518377441	0.136739	0.443637
1.128813245	0.073386	0.157978

SCR
NO AKT INHIB
0.60009
1.294563
1.324846

SUPPLEMENTARY FIGURES

SUPP 1B	
SCR	PDCD4 KO
2.67681509	0.54017035
1.2526999	0.60586491
0.14795349	0.24362005

SUPP 1C	
SCR	PDCD4 KO
0.129554	0.98206654
0.47559684	0.76731414
0.2037559	1.34366173

SUPP 1D	
SCR	PDCD4 KO
0.13193407	1.4526362
0.59344858	0.64315003
0.80329375	0.31279729

0.56096238	0.2989896
	0.48032225
1.61367607	0.78571951
1.59246631	0.53474154
1.48456895	0.20553316
0.99462795	0.20402515

0.48298146	0.4220873
0.10188478	0.23246131
0.13014406	0.20525984
0.20021964	0.84700534
0.07040332	0.3500632
0.24271356	0.2774145

0.60410299	0.37886907
0.07016849	0.10825724
0.10140526	0.3355842
	2.01136444
	0.77073481
0.26178974	0.20043314

SUPP 2B	
SCR	PDCD4 KO
0.341195	0.186119
1.477027	
0.982997	0.656367
0.897442	0.671155
1.747449	0.542404
1.057948	0.627255
0.974526	0.953741
0.386676	0.029818
1.008723	0.283435

SUPP 2C	
SCR	PDCD4 KO
0.447720	0.477503
0.605189	0.714651
0.573974	1.001416
0.731797	0.843764
0.339994	0.346530
0.472047	0.573758
0.509743	0.342218
0.397208	0.520462
0.378197	0.457726

SUPP 2D	
SCR	PDCD4 KO
0.075494	0.561669
0.630284	
0.094696	0.761133
0.065389	0.163275
	0.853121
0.665349	0.688169
0.881921	0.923694
0.939800	0.998904
0.749297	0.648581

1F	
SCR	PDCD4 KO
0.655051	1.269915
0.270941	1.152934
0.205746	0.582281

1G	
SCR	PDCD4 KO
0.493126	2.018271
0.376472	1.50429
0.42019	1.2

2E	
SCR	PDCD4 KO
0.203856	0.571975
0.308418	0.456048
0.264278	0.40925

3E	
SCR	PDCD4 KO
0.440814	0.166314
0.940683	0.485633
0.60953	0.506972

3G					
SCR	PDCD4 KO	SCR	PDCD4 KO	SCR	PDCD4 KO
0.946359	0.81709	0.231857	0.194679	0.50175	0.400892
1.120684	0.945943	0.244301	0.192644	0.346795	0.357796
0.783931	1.123035	0.214302	0.224069	0.374735	0.370683

4E	
SCR	PDCD4 KO
1.161083	1.450267
1.403408	1.580124
0.981887	1.480267

4F	
SCR	PDCD4 KO
1.676372	2.319471
1.082929	1.088022
0.320398	0.575868

4G			
10 H CHASE		24 H CHASE	
SCR	CTL	SCR	CTL
0.074	0.093	0.132	0.156
0.065	0.095	0.129	0.168
0.09	0.085	0.196	0.149
0.1	0.079	0.185	0.143
0.044	0.108	0.078	0.133

0.125	0.207	0.146	0.221

5D	
SCR	PDCD4 KO
100	77
100	78
100	74

5E	
SCR	PDCD4 KO
100	15
100	16
100	61
100	58

Fig 6B

SERINE		THREONINE		TYROSINE		ASPARTATE		GLUTAMATE	
SCR	PDCD4 KO	SCR	PDCD4 KO	SCR	PDCD4 KO	SCR	PDCD4 KO	SCR	PDCD4 KO
4.404388	2.448990		0.231255	1.273652	0.334963	1.641919	0.393771		0.131665
3.339194	2.744661	0.527724	0.650436	1.418633	0.384782	0.404949	0.308506	0.397408	0.125360
0.264661	0.177178	0.392855	0.306568	0.791252	0.718996	0.189982	0.108324	0.037221	0.241969
3.446624	1.697553	1.281530	0.451867	0.902273	0.601470	0.386412	0.178156	0.211432	0.015754
3.222605	2.992019	0.349251	0.680070	0.453655	0.525097	0.506812	0.308178	0.227456	0.126159
3.805185	0.180857	0.914107	0.337801	0.886122	0.585216	0.403444	0.019990	0.806209	0.016313
0.248860	0.330097	0.469545	0.093989	0.790052	0.910059	0.248659	0.221643	0.015305	0.038345
3.802657	3.709980	1.029266	0.943562	0.563944	0.520000	0.092002	0.398953	0.159723	0.073797
0.471581	1.738785	0.365977	0.493382	1.677110	0.697318	0.176604	0.412819	0.006707	0.118039

7D	
PDCD4 KO	PDCD4 KO
NO AKT INHIB	YES AKT INHIB
2.045356	1.167109
1.478278	1.978584
2.775935	1.384415

7E		
SCR	PDCD4 KO	PDCD4 KO
NO AKT INHIB	NO AKT INHIB	YES AKT INHIB
2.82475601	5.18359858	5.29647557
3.82465398	5.58642718	6.66047312
2.87077495	4.2066516	4.49243942

7F		
SCR	PDCD4 KO	PDCD4 KO
NO AKT INHIB	NO AKT INHIB	YES AKT INHIB
7.3828091	14.62816	10.8569789
8.124322	##########	12.6897085
7.652666	12.4359666	11.6127032

SUPP 1E	
SCR	PDCD4 KO
0.50469225	0.53849122
0.55571401	0.52895677
	0.50847623

0.58804209	

SUPP 2E	
SCR	PDCD4 KO
0.366960	0.731941
0.490891	0.747760
0.345445	0.529086
0.465273	0.704328
0.481295	0.585473
0.505420	0.766077
0.518932	0.504486
0.544736	0.669589
0.409356	0.848963

4H			
10 H CHASE		24 H CHASE	
SCR	CTL	SCR	CTL
0.578	0.723	0.454	0.436
0.553	0.759	0.458	0.501
0.793	0.651	0.556	0.459
0.995	0.603	0.528	0.385
0.229	0.735	0.123	0.147

4			
CONTROL		MG132	
SCR	CTL	SCR	CTL
	0.363897551	0.284169241	0.272151632
0.094314321	0.109094122		
0.110048626	0	0.202508276	0.208583214
0	0	0.715860156	
0.671638637	0.385048929	0.615769431	0.532041822

	0.743	0.779	0.233	0.234		0.093098358	0.45196511	0.401163736	0.793636984
						0			
						0.121445074			
						0.294843325	0.221642272	0.593755262	

LYSINE		ARGININE		HISTIDINE		GLYCINE		ALANINE	
SCR	PDCD4 KO	SCR	PDCD4 KO	SCR	PDCD4 KO	SCR	PDCD4 KO	SCR	PDCD4 KO
	0.020630	1.437047	0.053189				0.013933		0.056886
0.635901	0.021865	0.037440	0.050074	1.782560		0.100517	0.116607	0.032519	0.031163
0.890927	0.481395	0.042511	0.007457	1.821580	1.316193	0.125259	0.058548	0.183438	0.016615
1.434638	1.098590	0.019566	0.040237	5.850467	1.915281	0.190749	0.043333	0.033263	0.007359
1.283252	1.112040	0.072256	0.078212			0.158047	0.031880	0.083693	0.029505
1.428932	1.214336	0.195141	0.084059			0.361428	0.087560	1.504833	0.334263
1.467293	1.485668	0.087385	0.011520	2.645659	1.681902	0.094256	0.091852	0.019746	0.018880
1.413905	1.502539	0.073406	0.163489			0.084583	0.077628	0.092178	0.035219
0.888112	0.499711	0.041620	0.195807	1.146531	0.591738	0.036080	0.550523	0.024108	0.087538

[illegible]

J			
STARVED		MG132 + STARVED	
SCR	CTL	SCR	CTL
		0.568656962	0.402943235
0.046578186	0.082258127	0.222846586	0.263023385
0.158281971	0.144563043	0.311504177	0.212071829
		2.027008692	
0.252027408	0.395297702	0.845327791	0.473647774

0.414825571	0.586202971	1.052103359	0.883936492
		1.055119229	
0.638306678	0.201039976		0.624883928
0.2469505	0.200134642	1.056008956	

PROLINE		PHENYLALANINE	
SCR	PDCD4 KO	SCR	PDCD4 KO
3.123636	0.814808	0.526216	0.137923
5.143875	0.734775	0.247322	0.142086
3.882191	0.338404	0.059057	0.198042
5.145282	3.163252	0.272141	0.218703
5.109293	0.374227	0.305290	0.220301
3.777822	0.816690	0.176442	0.213433
5.176077	0.518560	0.285718	0.284214
5.348188	0.863696	0.309440	0.322832
4.294048	1.032068	0.049707	0.065731

