# Supplementary material for: Myofibrillar protein accumulation but reduced protein synthesis in PDCD4-depleted myotubes
Source: PLoS One. 2026 Mar 19;21(3):e0345305. doi: 10.1371/journal.pone.0345305 (PMC13001914; doi:10.1371/journal.pone.0345305)
Supplement: S2 File — (PDF) [file pone.0345305.s005.pdf]

Mora et al. Myofibrillar protein accumulation but reduced protein synthesis in PDCD4-depleted myotubes

Original Blots

Figure 1A – PDCD4

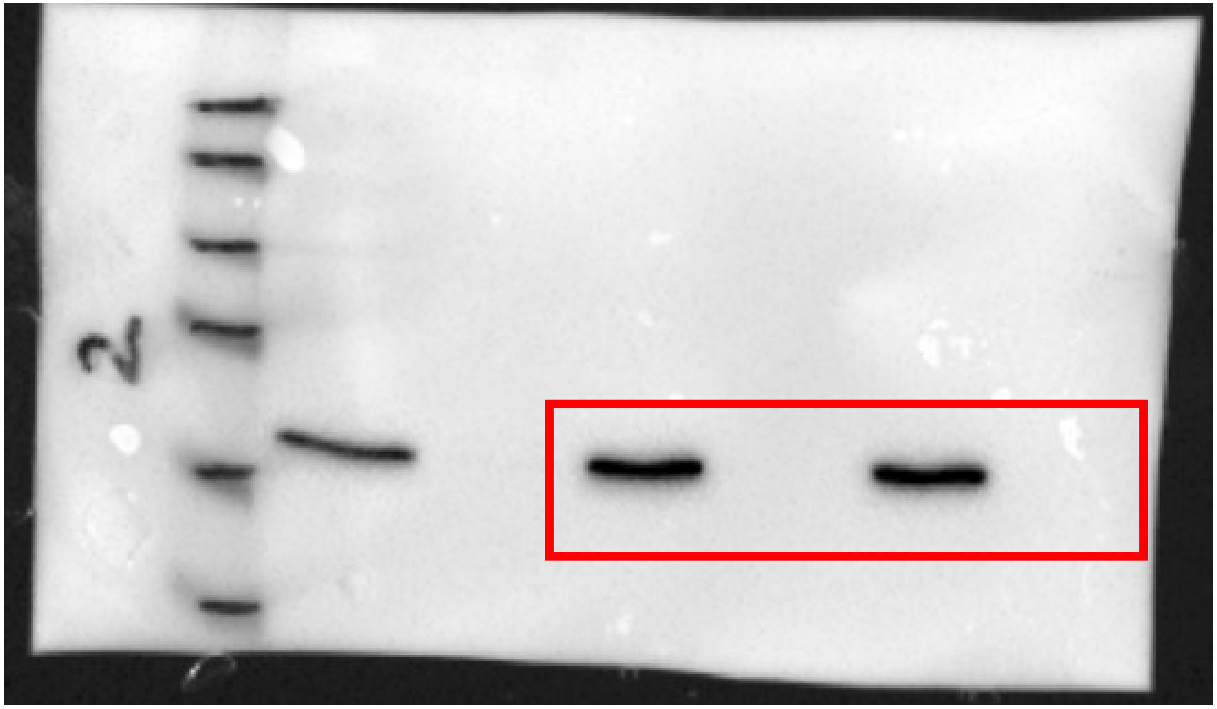

Blot Order

SCR – PDCD4 siRNA – SCR – PDCD4 siRNA

Figure 1D – MHC

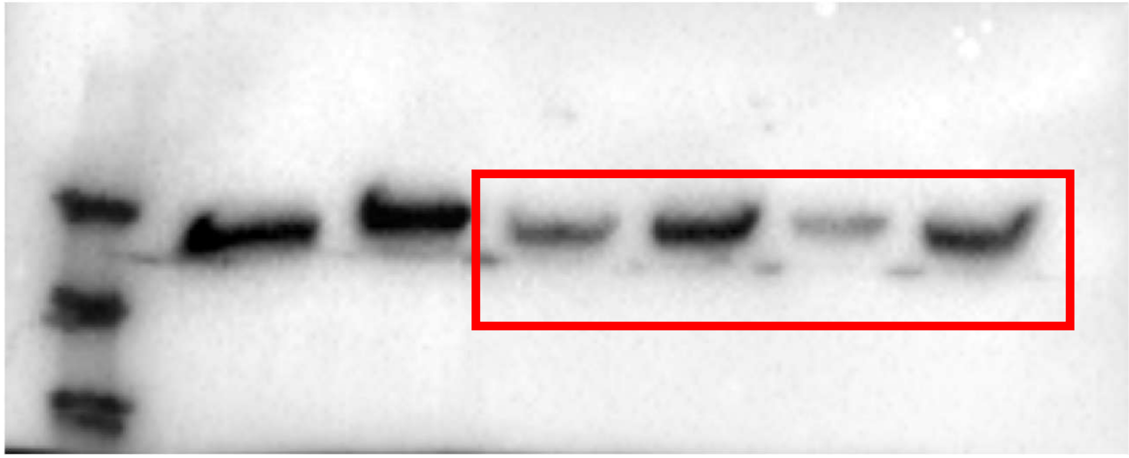

Blot Order

SCR – PDCD4 siRNA – SCR – PDCD4 siRNA

Figure 1D – Troponin

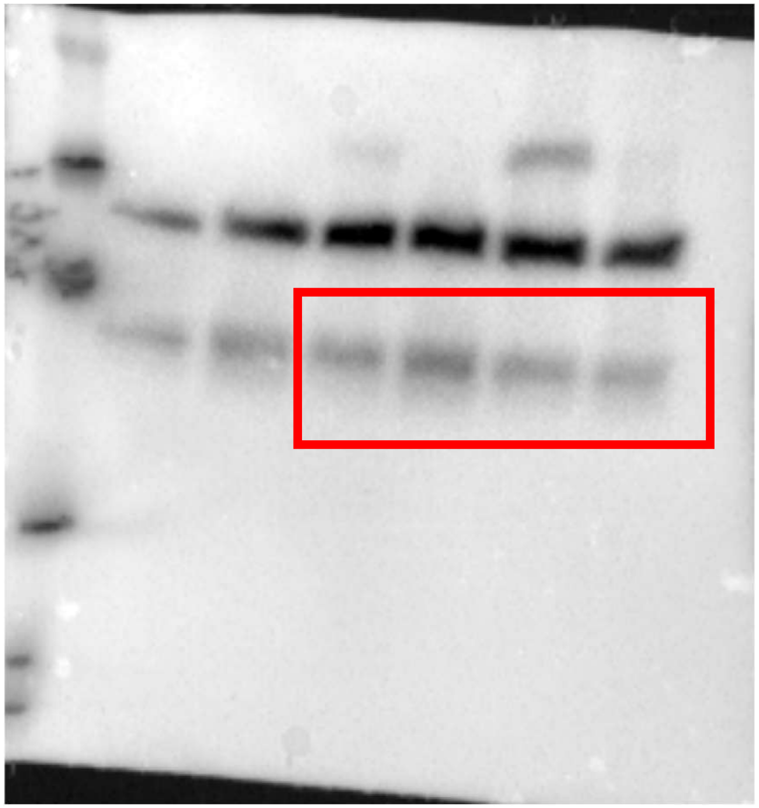

Blot Order

SCR – PDCD4 siRNA – SCR – PDCD4 siRNA

Figure 1D – Tropomyosin

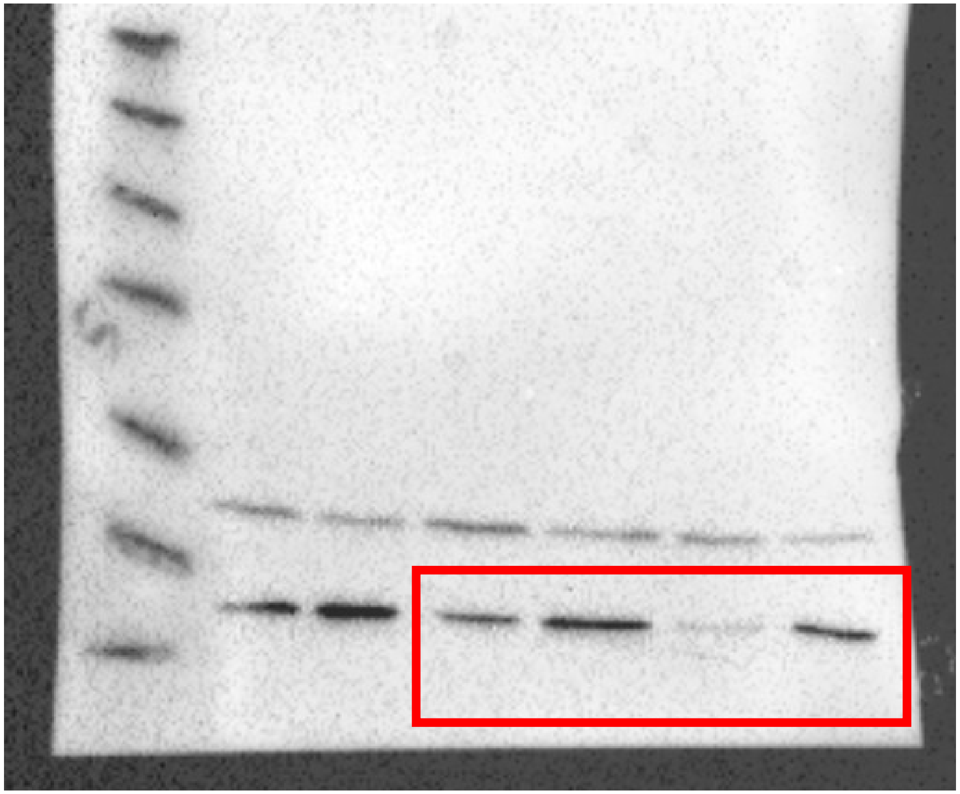

Blot Order

SCR – PDCD4 siRNA – SCR – PDCD4 siRNA

Figure 2A – MHC

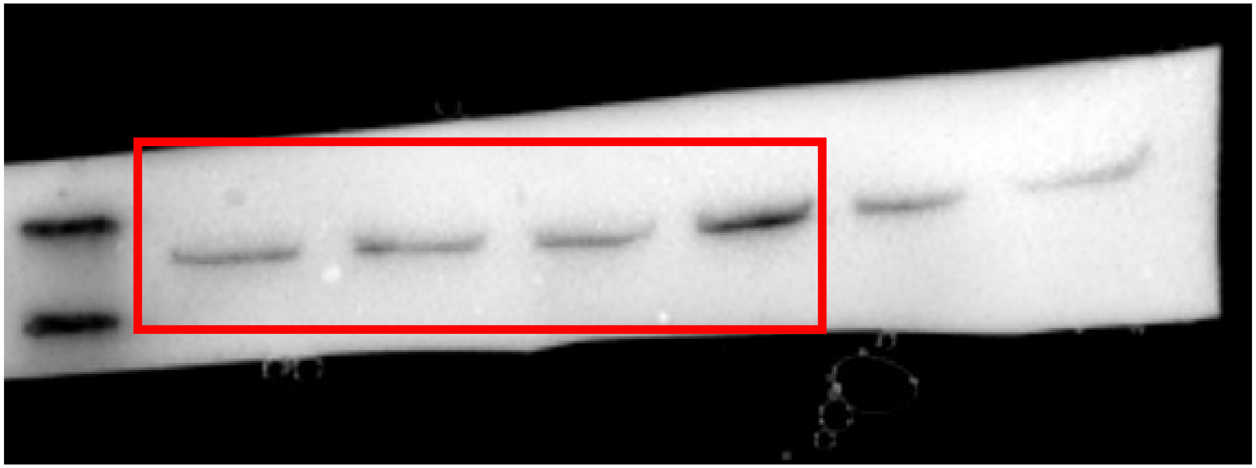

Blot Order

SCR – PDCD4 siRNA – SCR – PDCD4 siRNA

Figure 2A – PDCD4

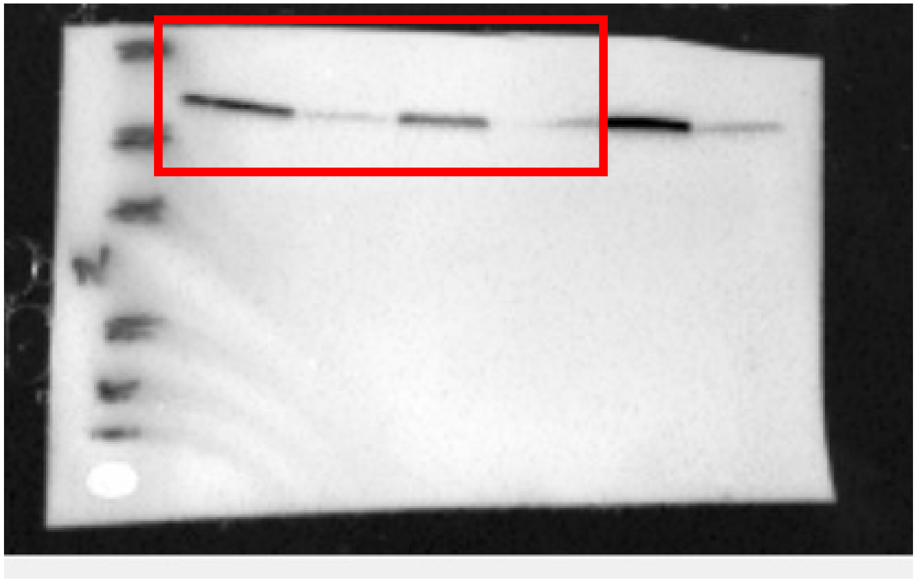

Blot Order

SCR – PDCD4 siRNA – SCR – PDCD4 siRNA

Figure 2A – Troponin

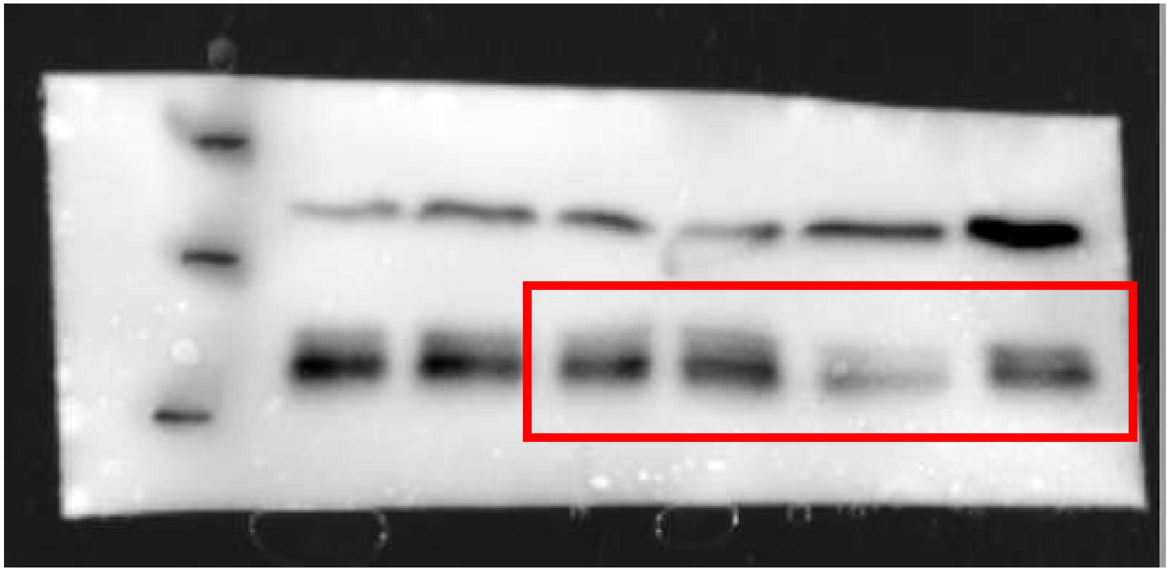

Blot Order

SCR – PDCD4 siRNA – SCR – PDCD4 siRNA

Figure 2A – Tropomyosin

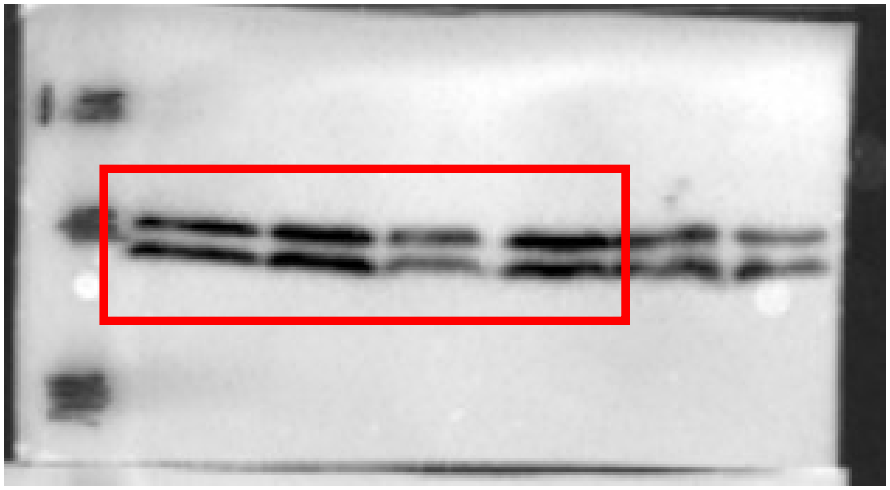

Blot Order

SCR – PDCD4 siRNA – SCR – PDCD4 siRNA

Figure 3A – p-Akt

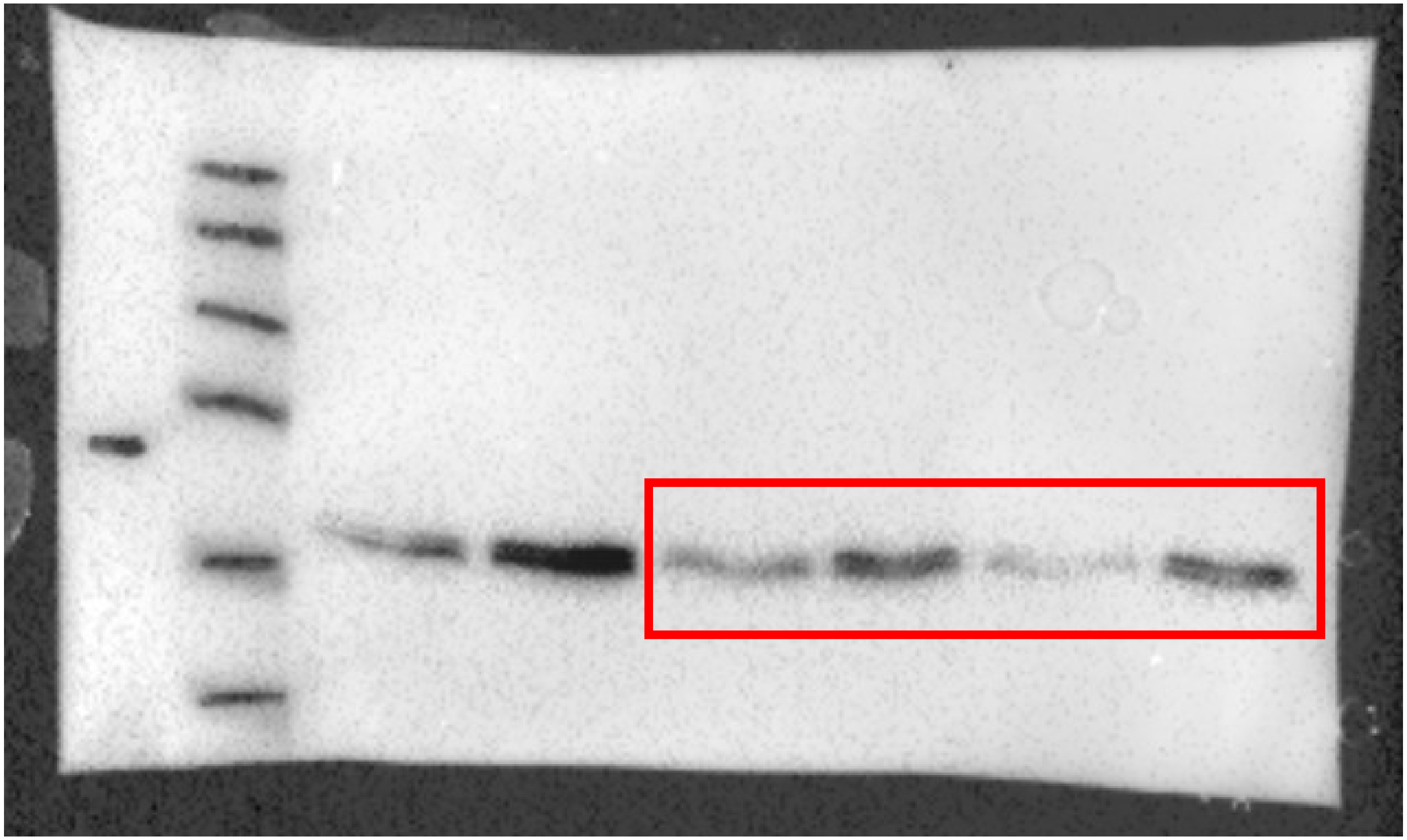

Blot Order

SCR – PDCD4 siRNA – SCR – PDCD4 siRNA

Figure 3A – p-S6K1

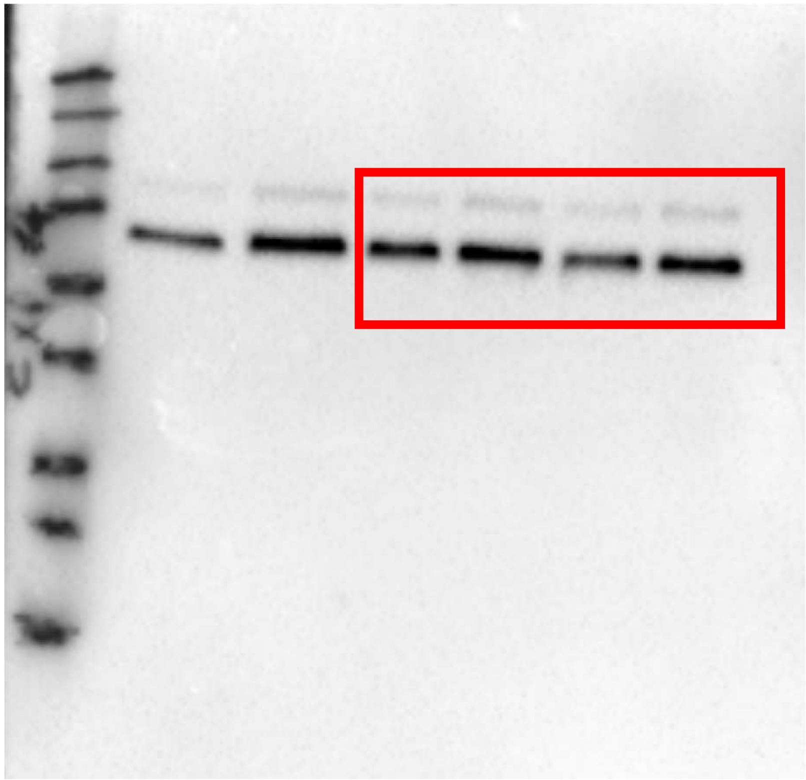

Blot Order

SCR – PDCD4 siRNA – SCR – PDCD4 siRNA

Figure 3A – p-S6

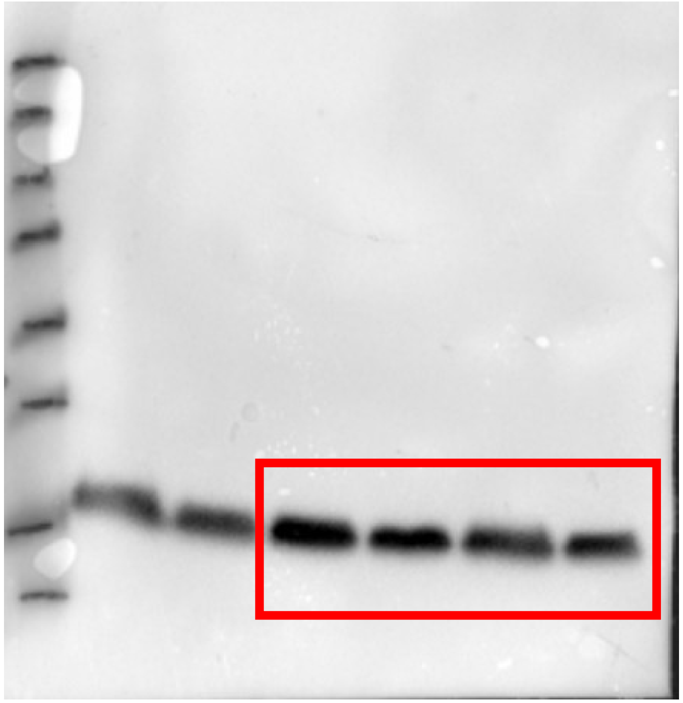

Blot Order

SCR – PDCD4 siRNA – SCR – PDCD4 siRNA

Figure 3A – p-4EBP1

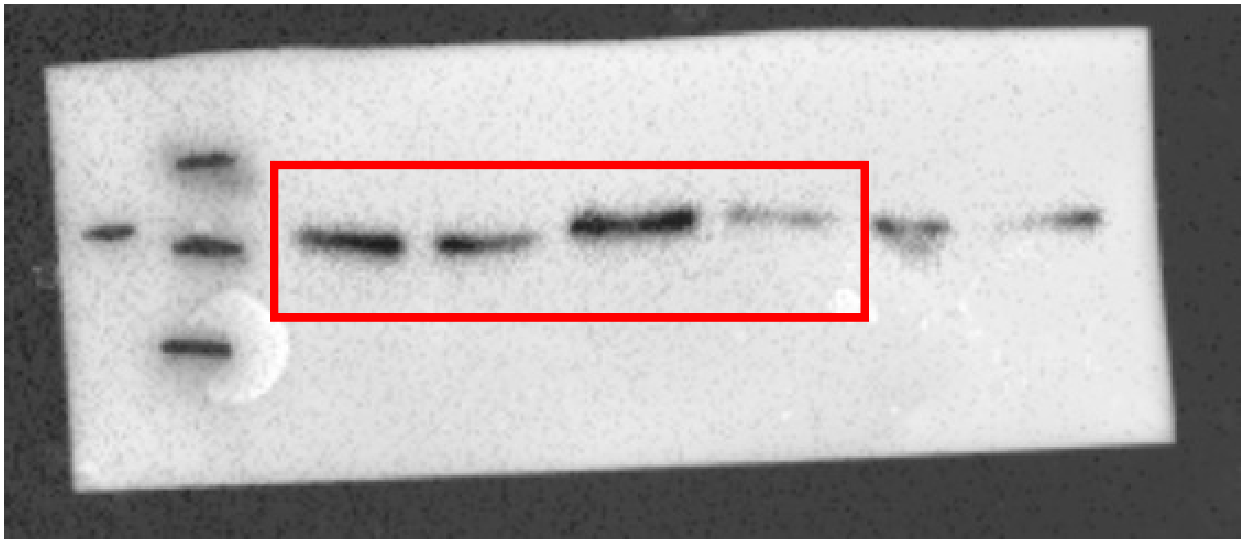

Blot Order

SCR – PDCD4 siRNA – SCR – PDCD4 siRNA

Figure 3F – S6K1

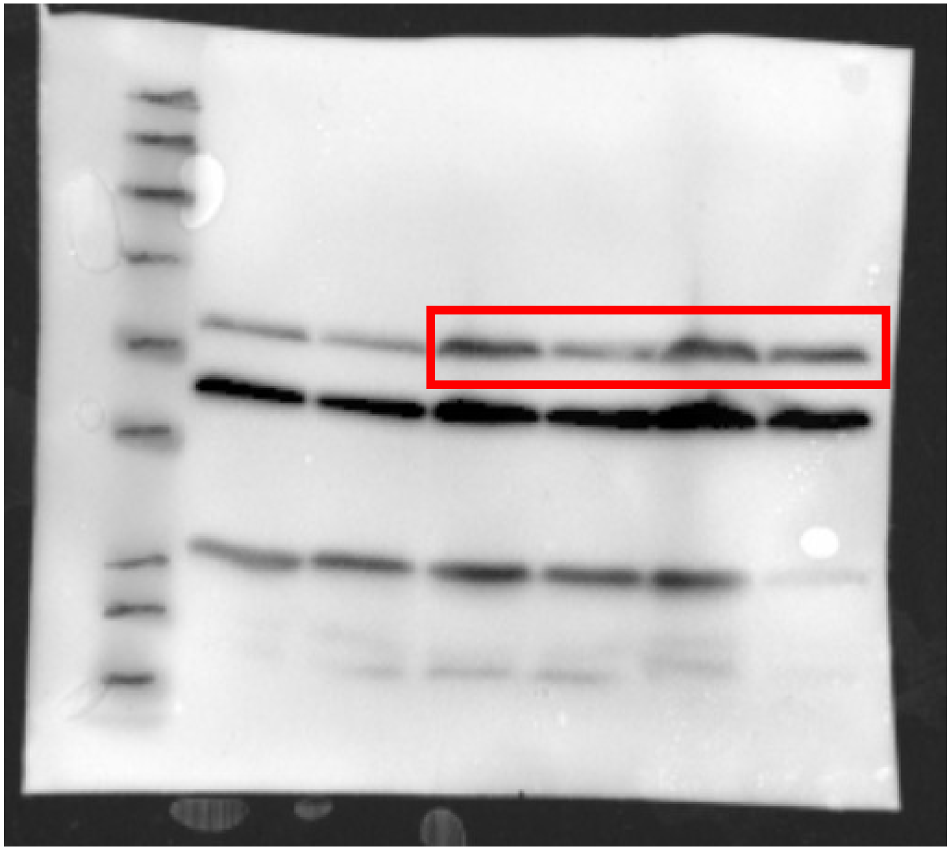

Blot Order

SCR – PDCD4 siRNA – SCR – PDCD4 siRNA

Figure 3F – Total AKT

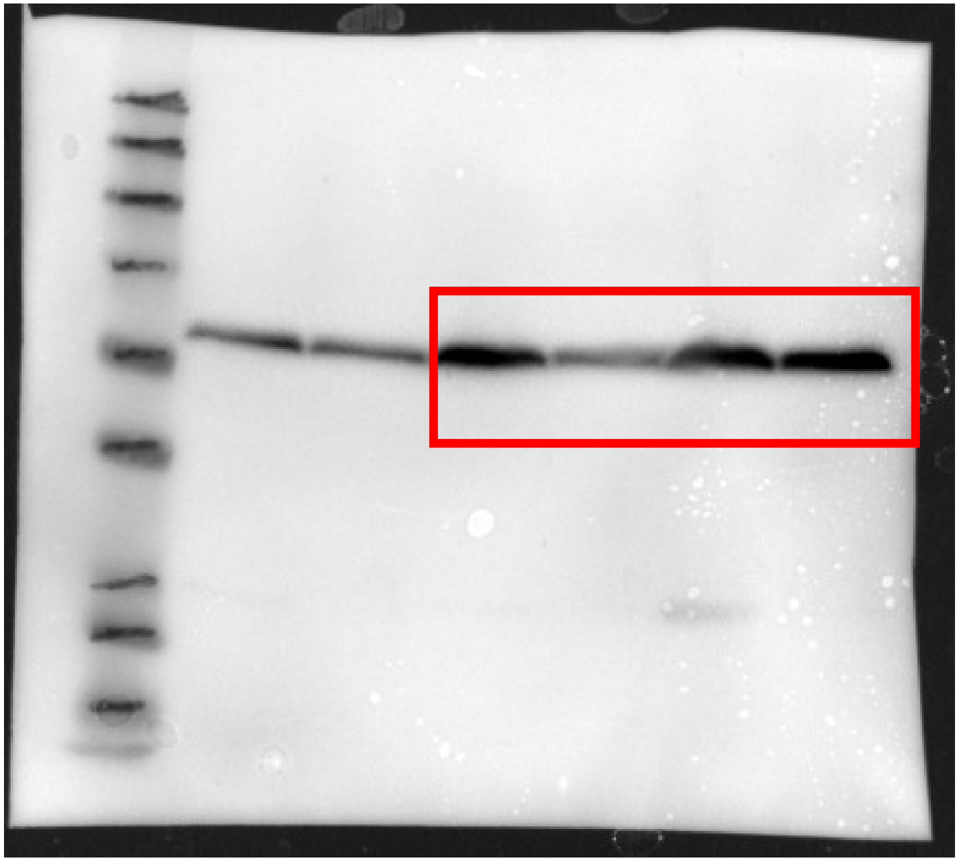

Blot Order

SCR – PDCD4 siRNA – SCR – PDCD4 siRNA

Figure 3F – Total S6

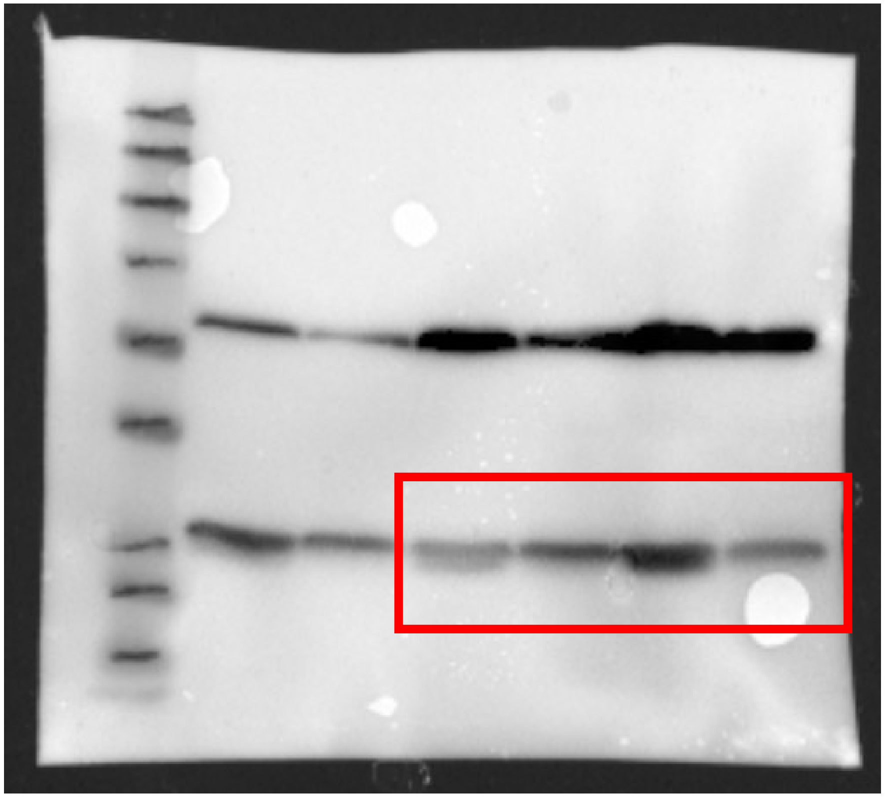

Blot Order

SCR – PDCD4 siRNA – SCR – PDCD4 siRNA

Figure 3F – Puromycin

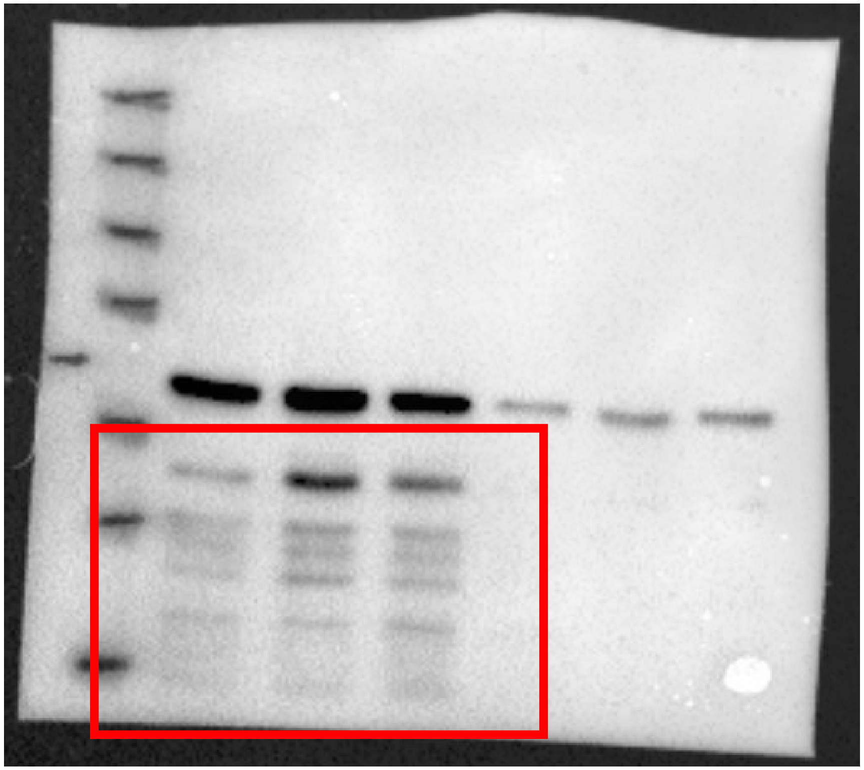

Blot Order

SCR – SCR – SCR – PDCD4 siRNA – PDCD4 siRNA – PDCD4 siRNA

Figure 3F – PDCD4

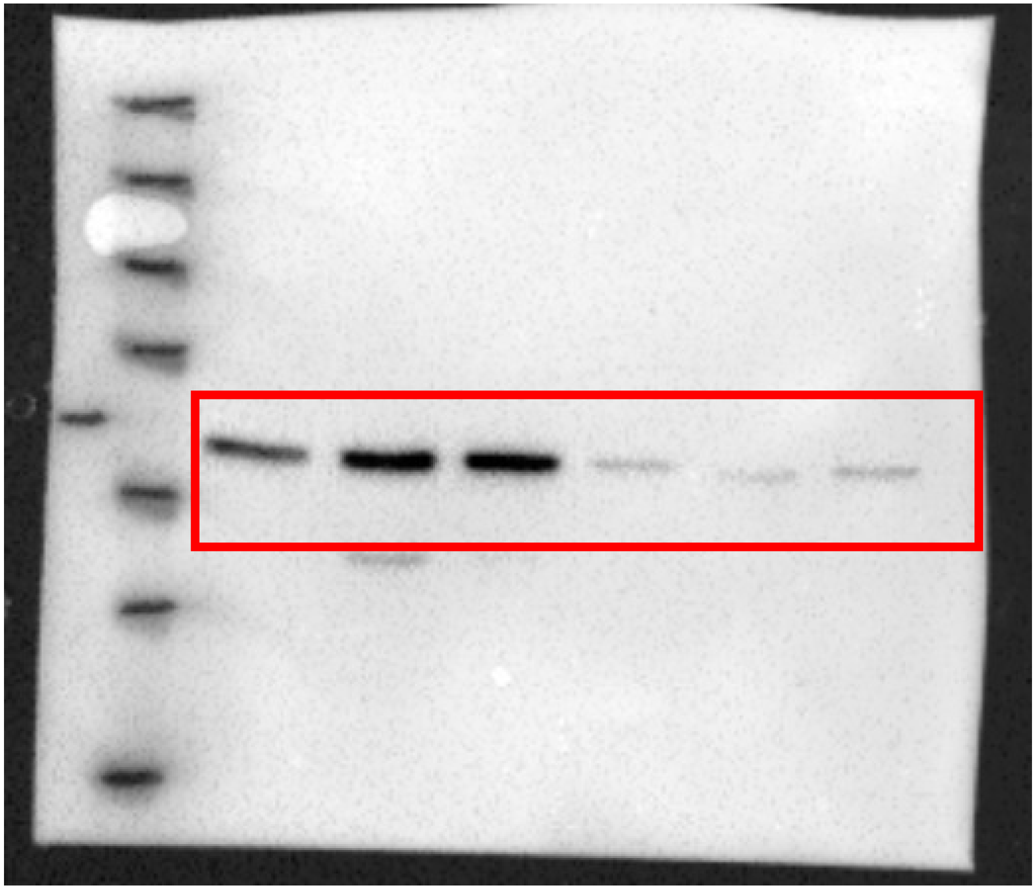

Blot Order

SCR – SCR – SCR – PDCD4 siRNA – PDCD4 siRNA – PDCD4 siRNA

Figure 3F – Ponceau S

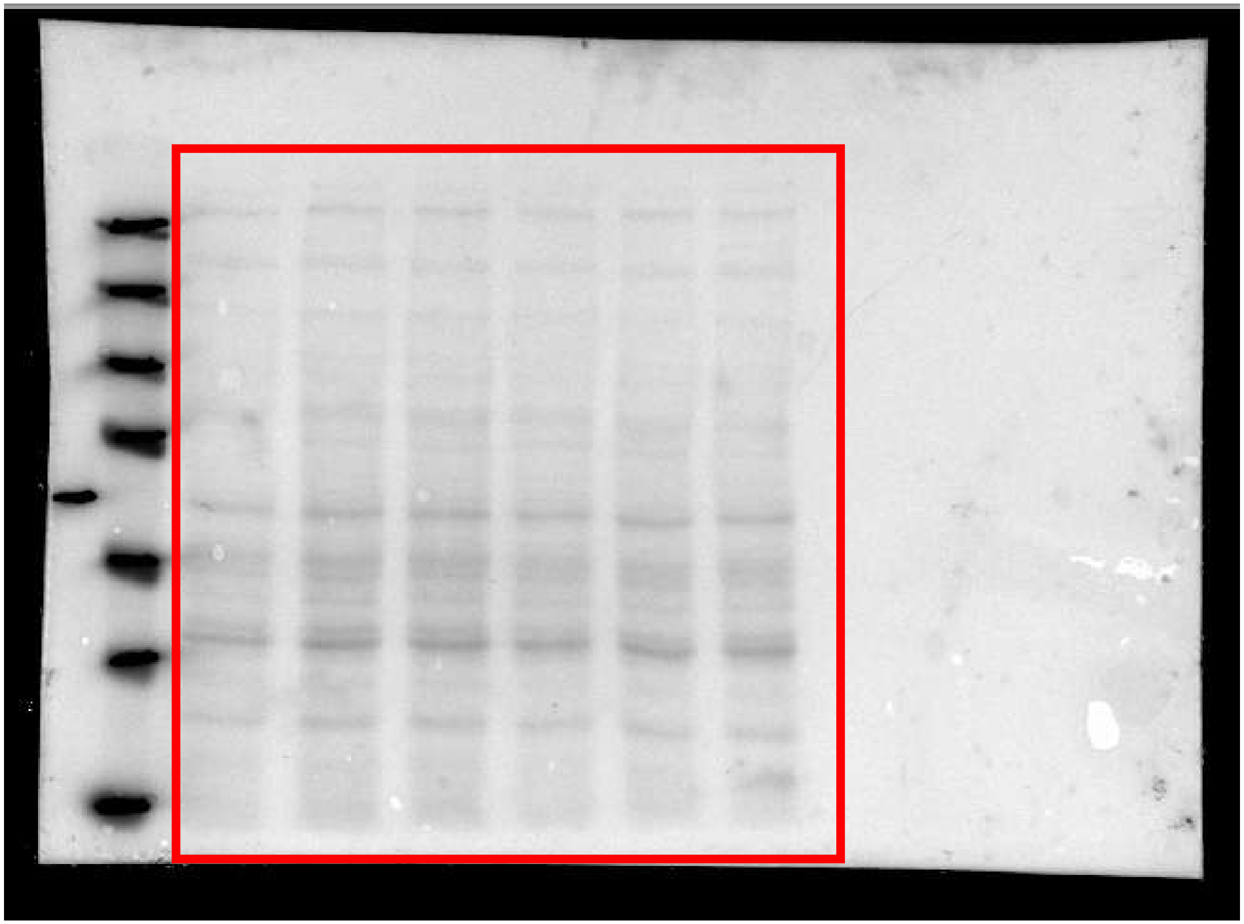

Blot Order

SCR – SCR – SCR – PDCD4 siRNA – PDCD4 siRNA – PDCD4 siRNA

Figure 4A – MuRF1

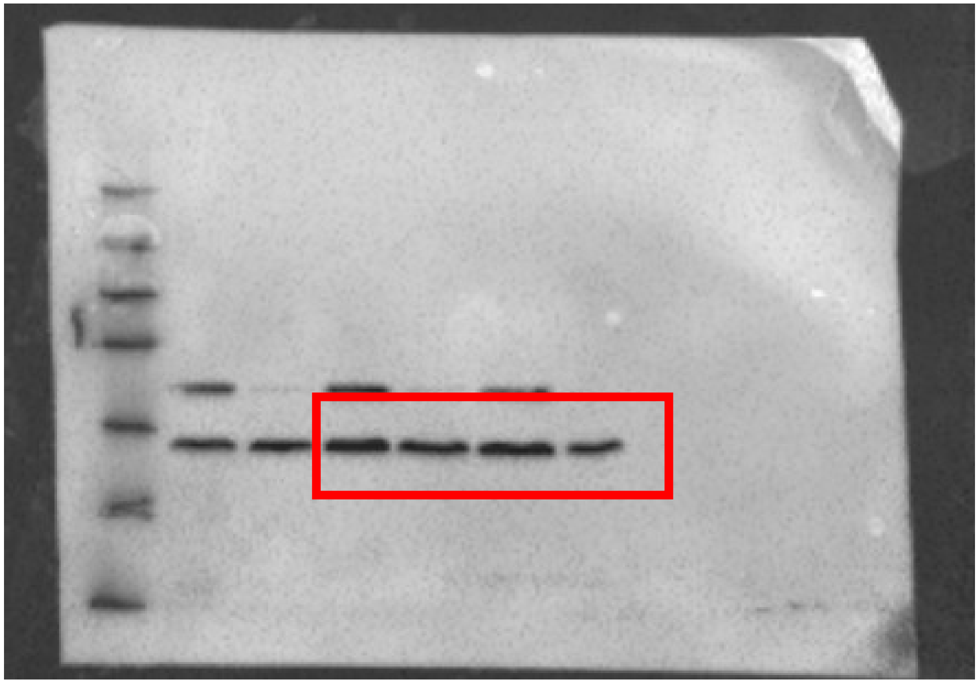

Blot Order

SCR – PDCD4 siRNA – SCR – PDCD4 siRNA

Figure 4A – Beclin-1

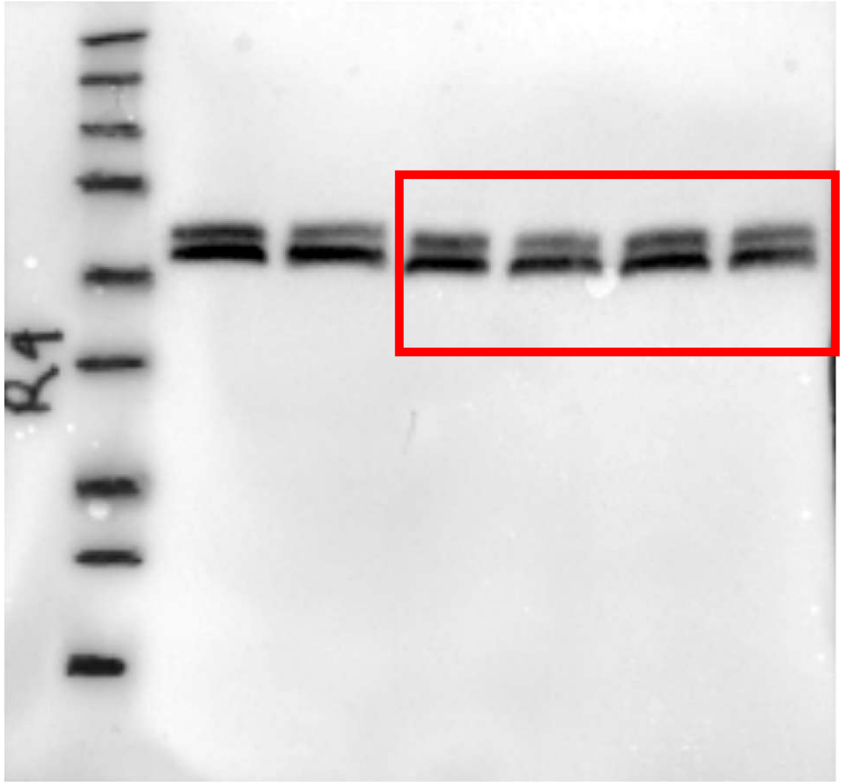

Blot Order

SCR – PDCD4 siRNA – SCR – PDCD4 siRNA

Figure 4A – LC3B I/II

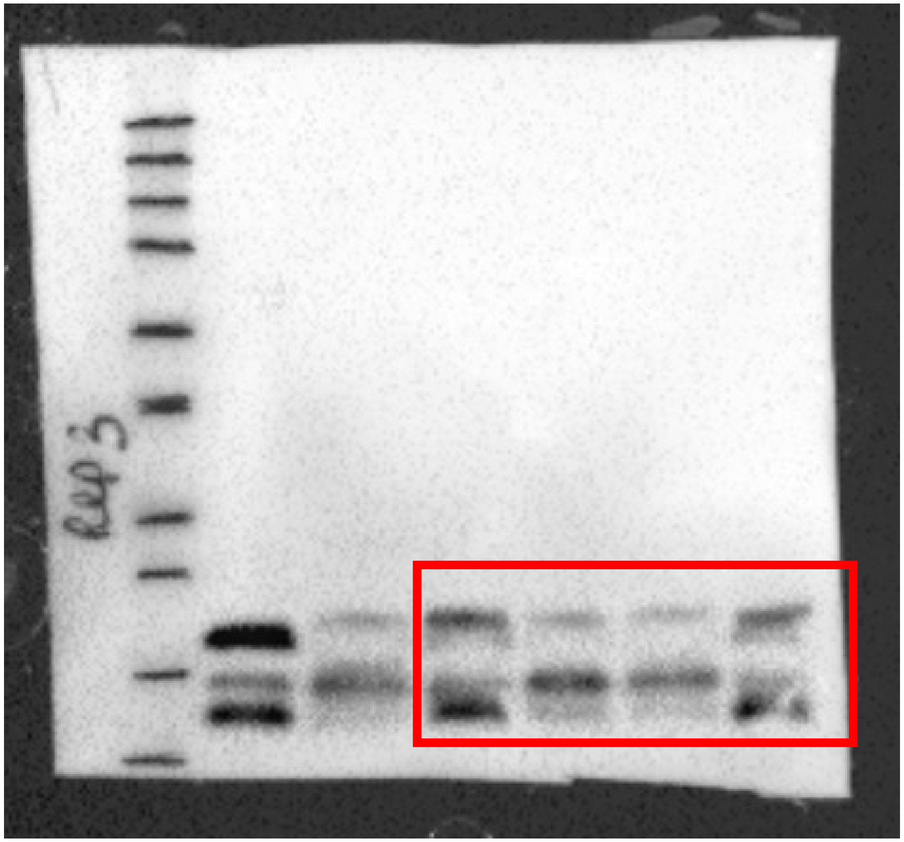

Blot Order

SCR – PDCD4 siRNA – SCR – PDCD4 siRNA

Figure 4A – P62

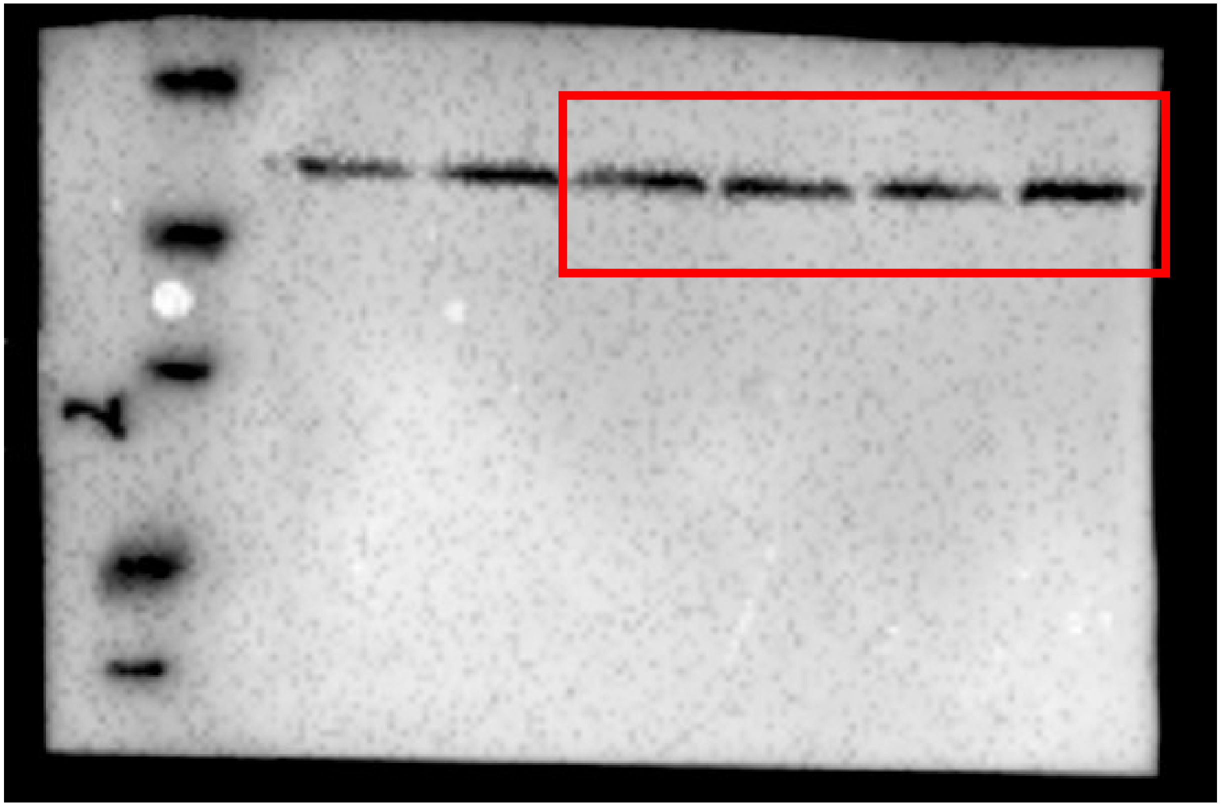

Blot Order

SCR – PDCD4 siRNA – SCR – PDCD4 siRNA

Figure 4I – Ubiquitin

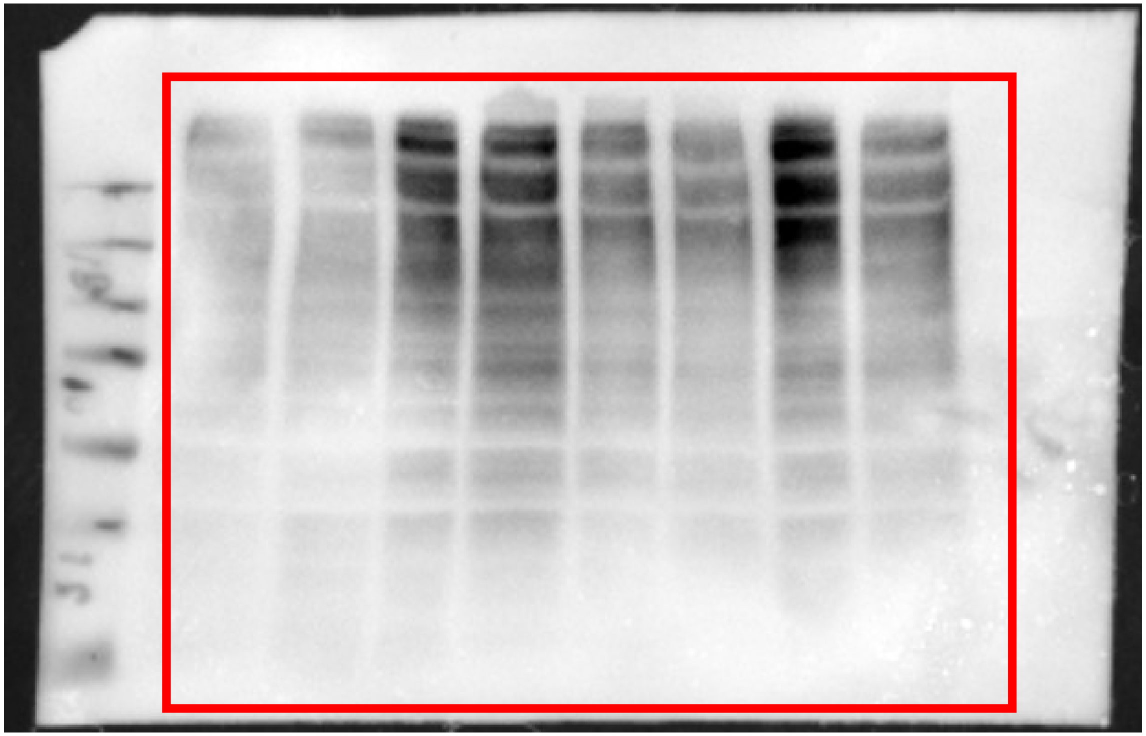

Blot Order

Control (x2) – MG132 (x2) – Starved (x2) – MG132 Starved (2)

PDCD4 siRNA: - + - + - + - +

Figure 4I – PonceauS

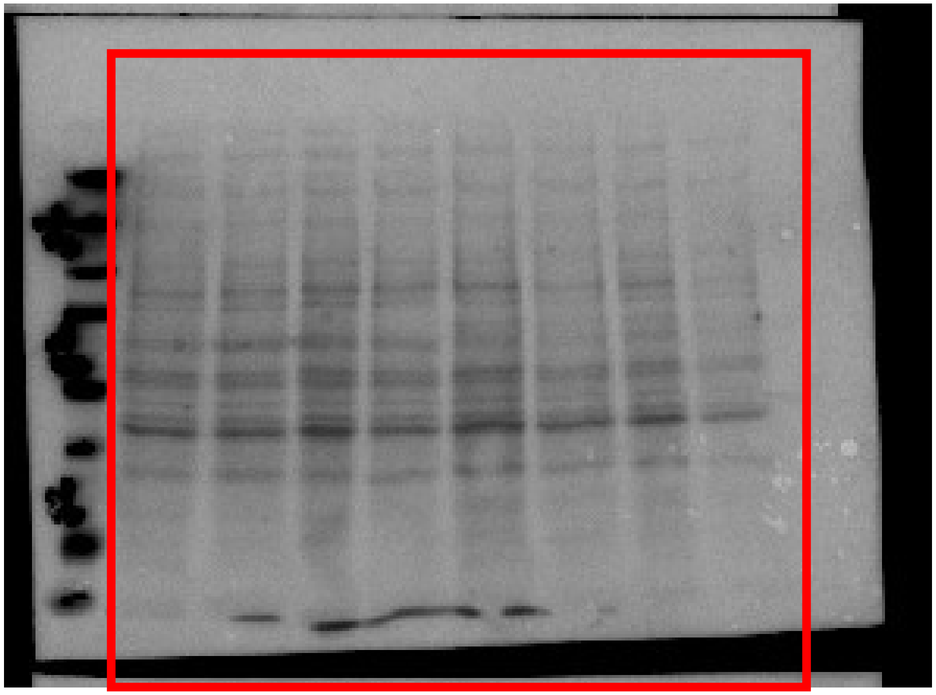

<u>Blot Order</u>

Control (x2) – MG132 (x2) – Starved (x2) – MG132 Starved (2)

PDCD4 siRNA: - + - + - + - +

Figure 4I – Puromycin

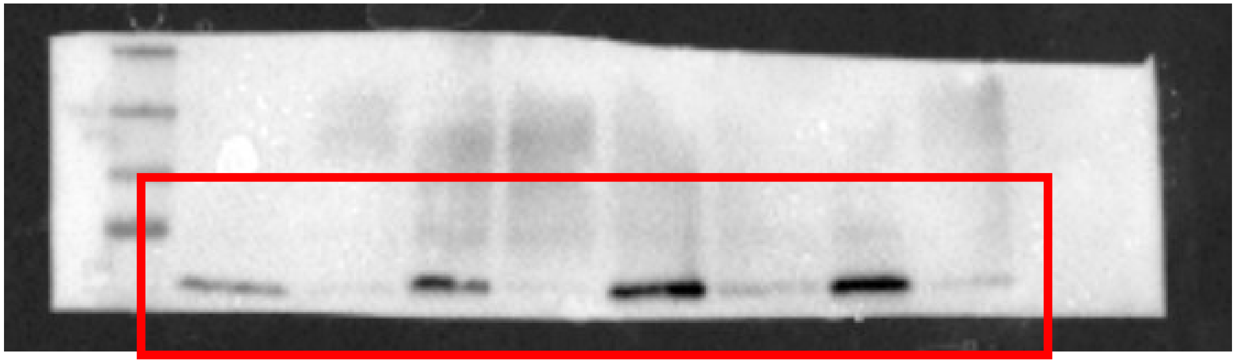

Blot Order

Control (x2) – MG132 (x2) – Starved (x2) – MG132 Starved (2)

PDCD4 siRNA: - + - + - + - +

Figure 7 – p-AKT

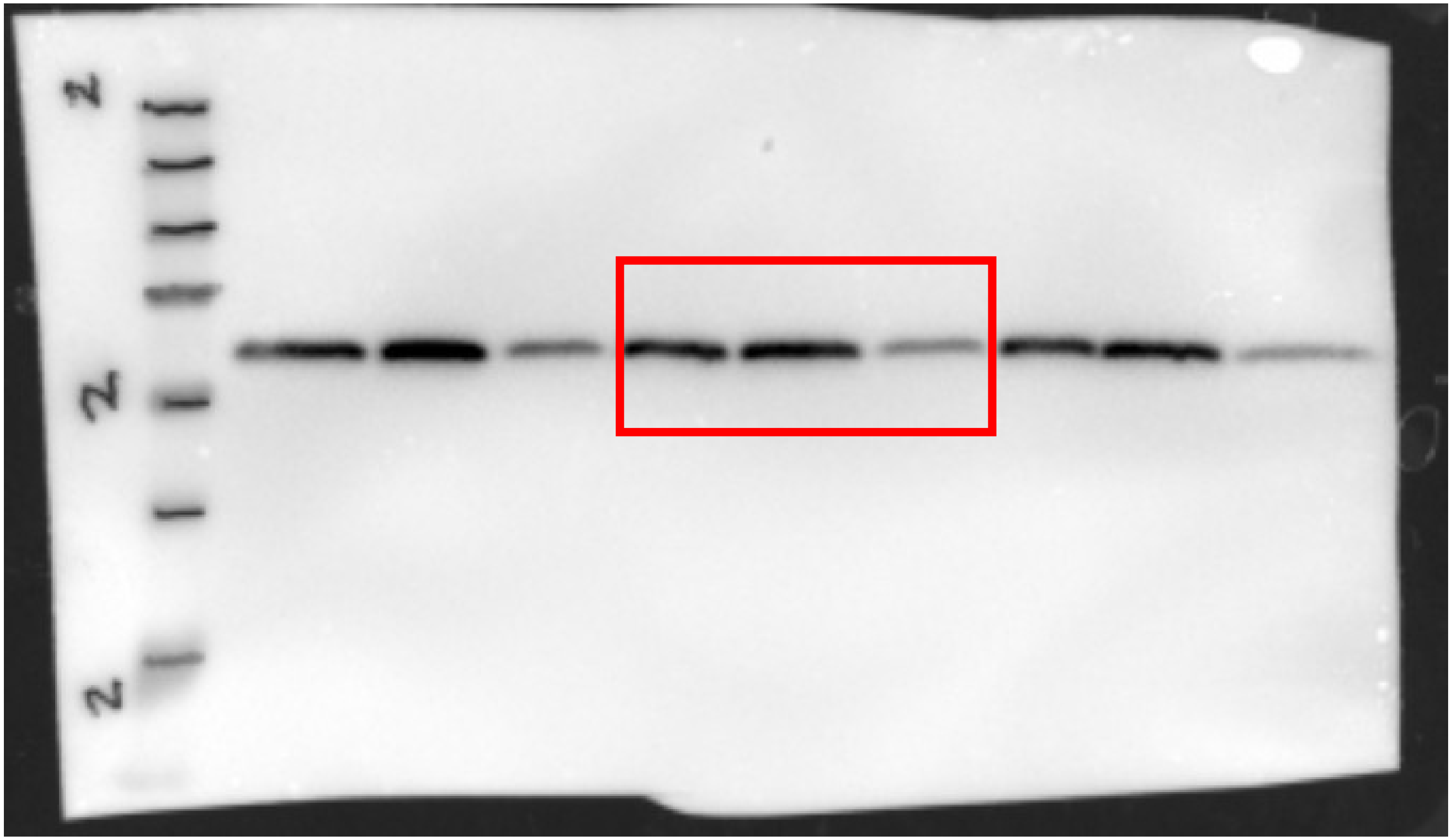

PDCD4 siRNA:	-	+	+	-	+	+	-	+	+
Akt Inhibitor:	-	-	+	-	-	+	-	-	+

Figure 7 – PDCD4

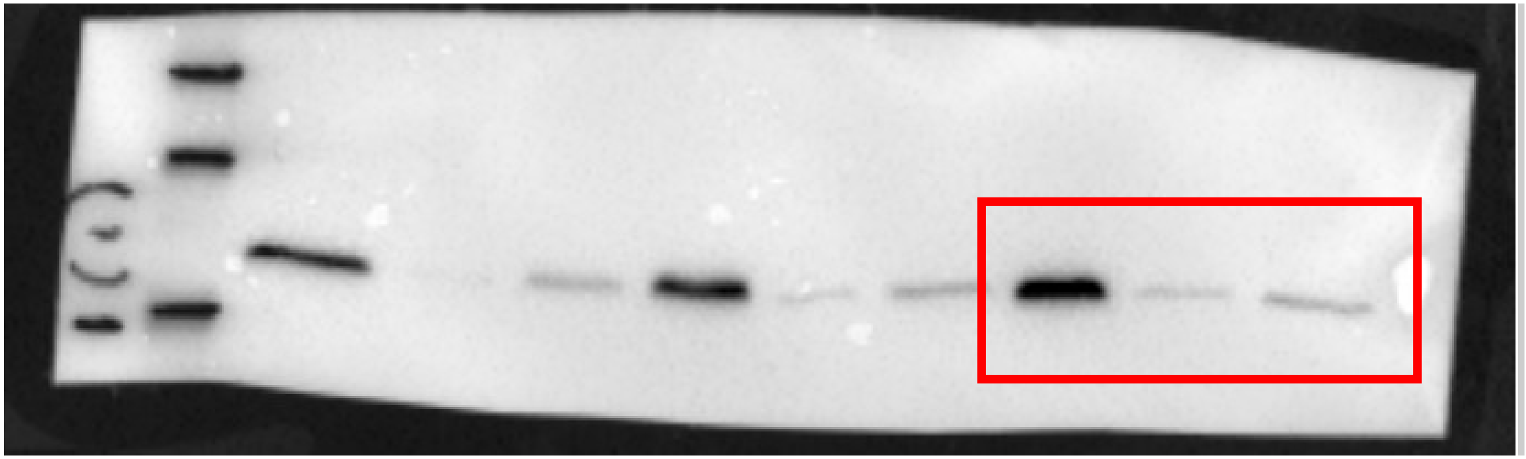

PDCD4 siRNA:	-	+	+	-	+	+	-	+	+
Akt Inhibitor:	-	-	+	-	-	+	-	-	+

Figure 7 – MHC-1

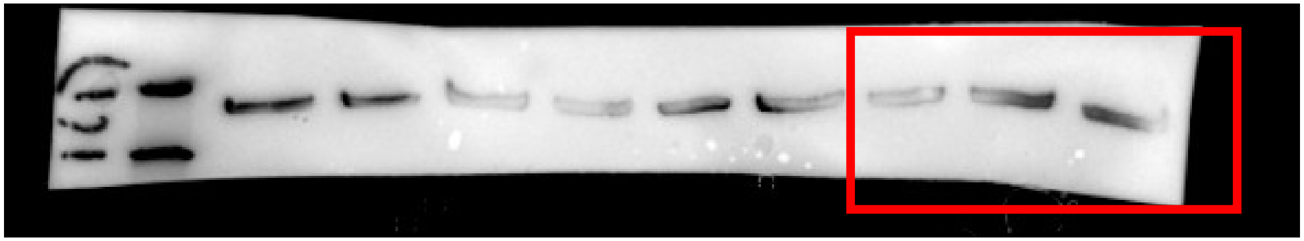

PDCD4 siRNA:	-	+	+	-	+	+	-	+	+
Akt Inhibitor:	-	-	+	-	-	+	-	-	+

Figure 7 – Troponin

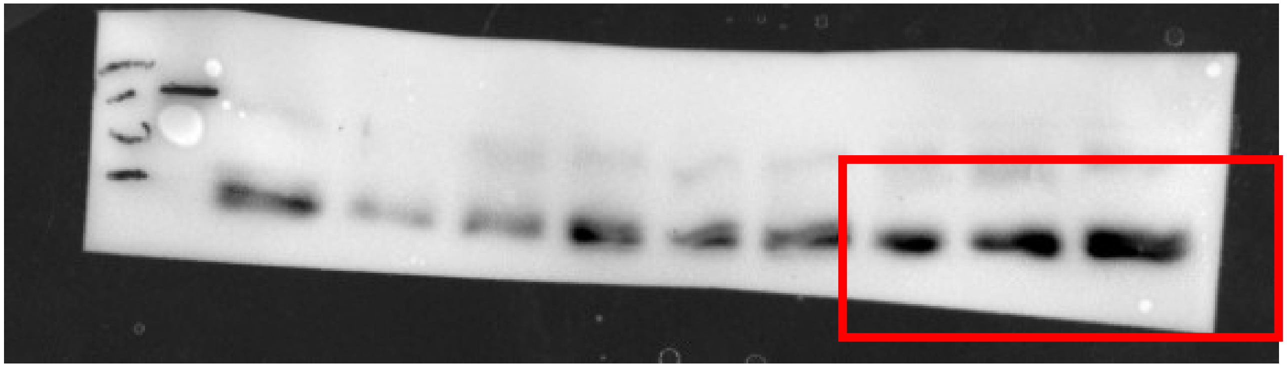

PDCD4 siRNA:	-	+	+	-	+	+	-	+	+
Akt Inhibitor:	-	-	+	-	-	+	-	-	+

Figure 7 – Tropomyosin

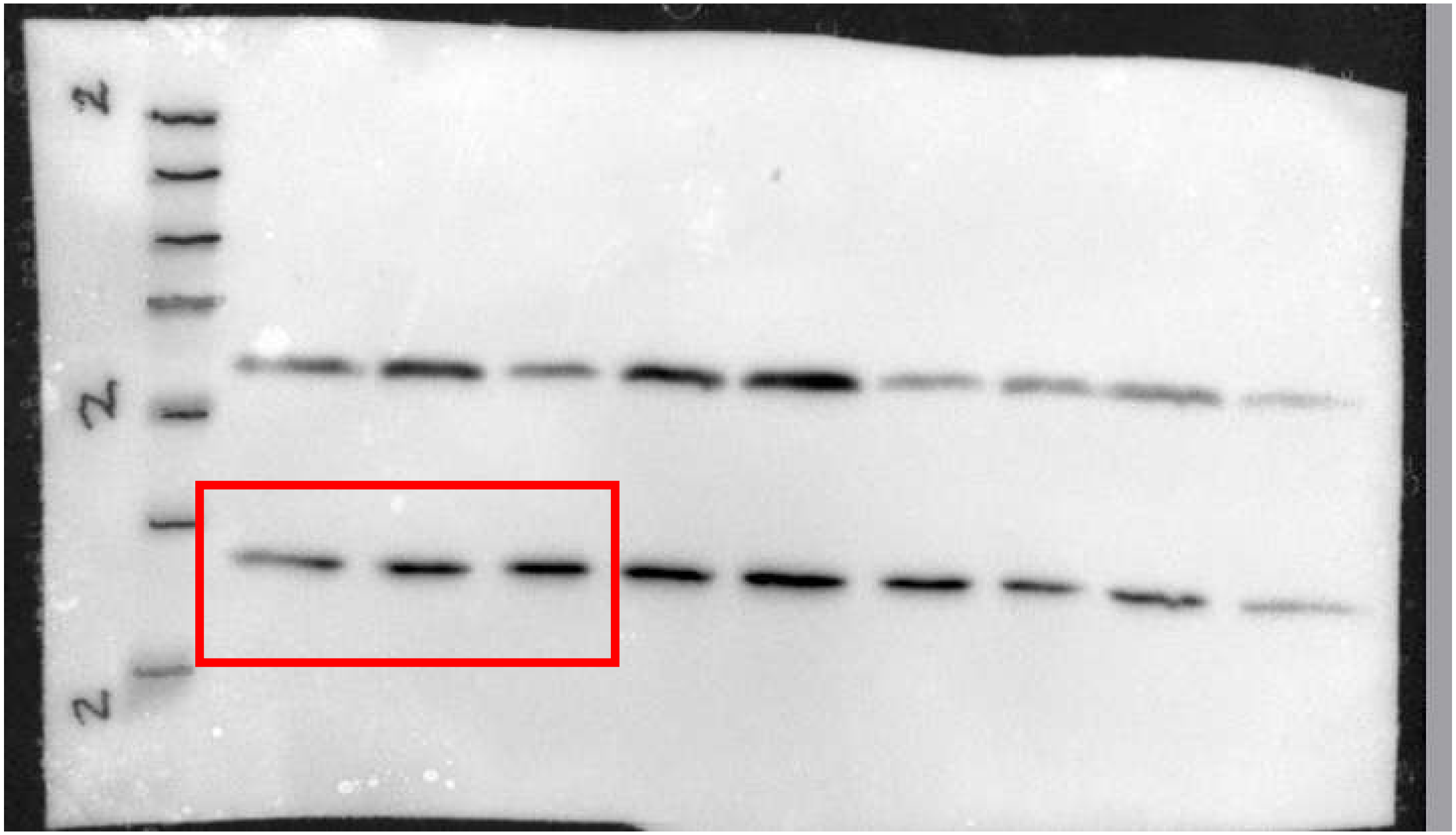

PDCD4 siRNA:	-	+	+	-	+	+	-	+	+
Akt Inhibitor:	-	-	+	-	-	+	-	-	+

L6 – MHC-1, related to S2A Fig

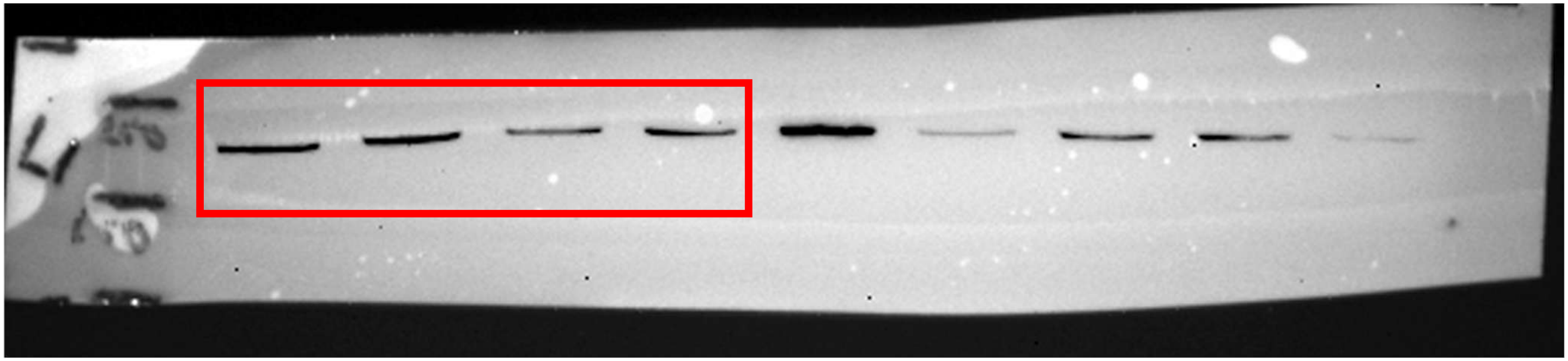

Blot Order

SCR – PDCD4 siRNA – SCR – PDCD4 siRNA

L6 – Troponin, related to S2A Fig

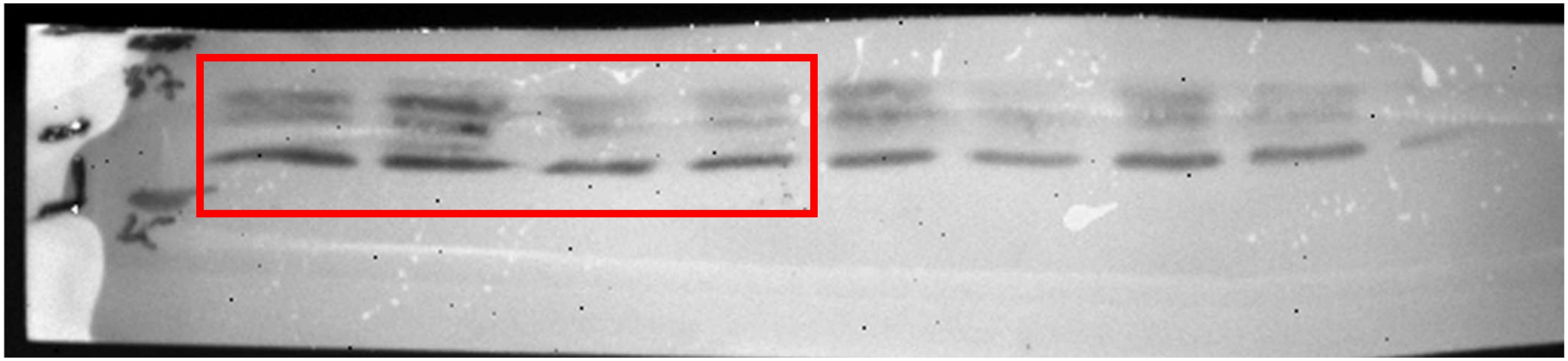

Blot Order

SCR – PDCD4 siRNA – SCR – PDCD4 siRNA

L6 – PDCD4, related to S2A Fig

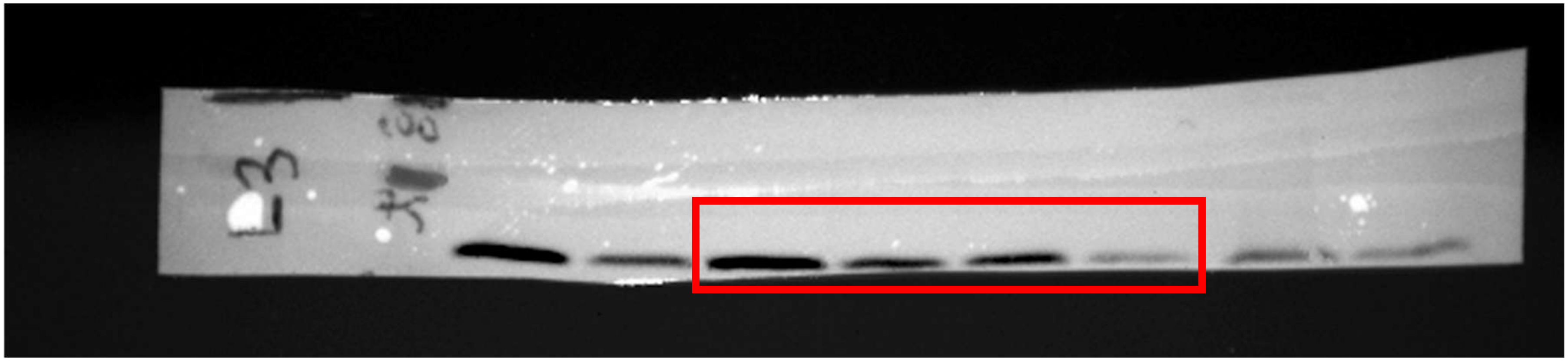

Blot Order

SCR – PDCD4 siRNA – SCR – PDCD4 siRNA

L6 – Tropomyosin, related to S2A Fig

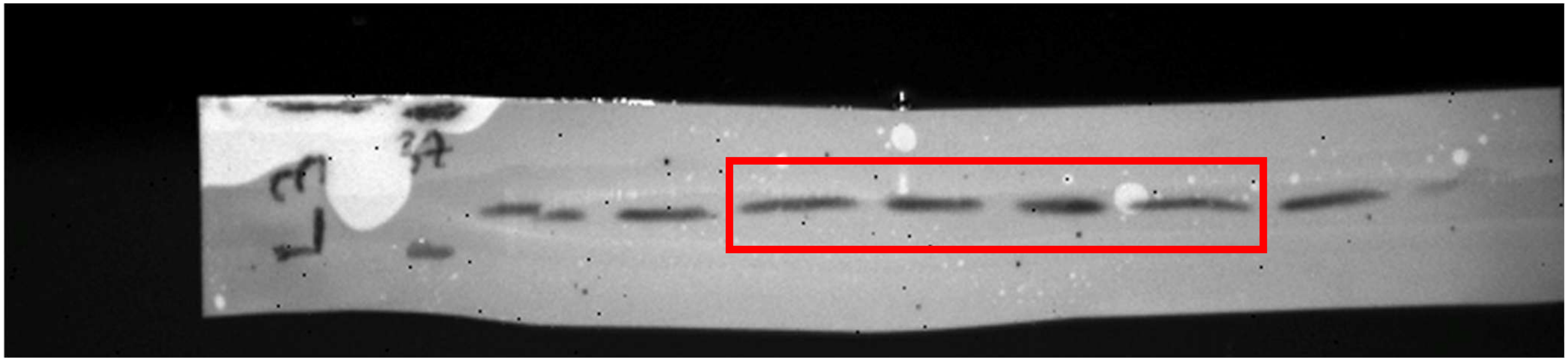

Blot Order

SCR – PDCD4 siRNA – SCR – PDCD4 siRNA

C2C12 – PDCD4 related to S3A Fig

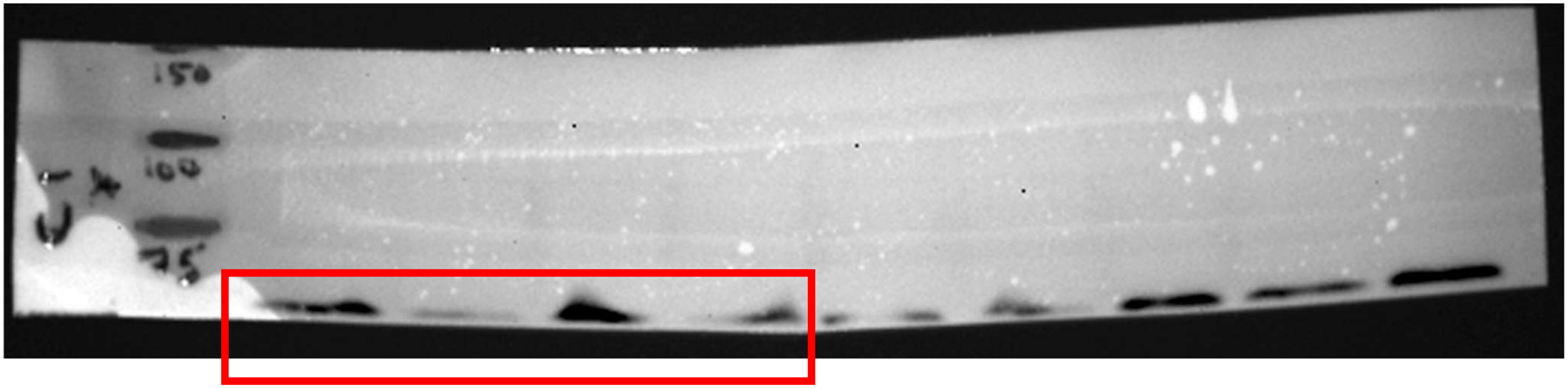

Blot Order

SCR – PDCD4 siRNA – SCR – PDCD4 siRNA

C2C12 – Troponin, related to S3A Fig

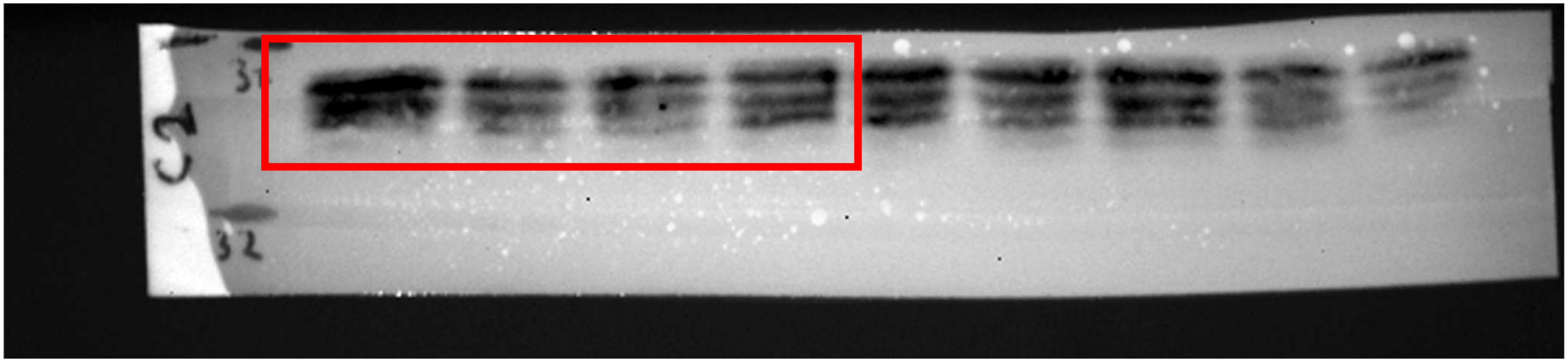

<u>Blot Order</u>

SCR – PDCD4 siRNA – SCR – PDCD4 siRNA

C2C12 – MHC-1, related to S3A Fig

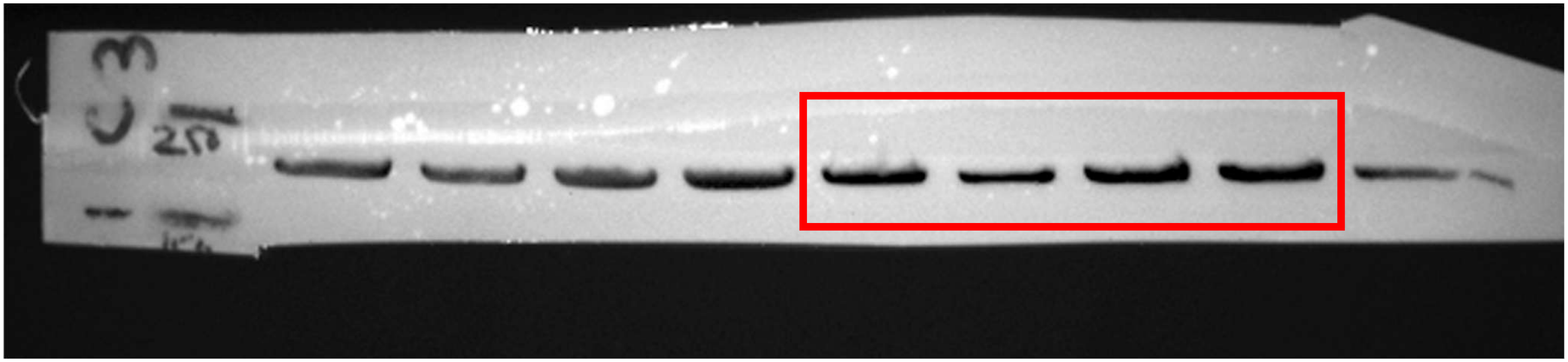

Blot Order

SCR – PDCD4 siRNA – SCR – PDCD4 siRNA

C2C12 – Tropomyosin, related to S3A Fig

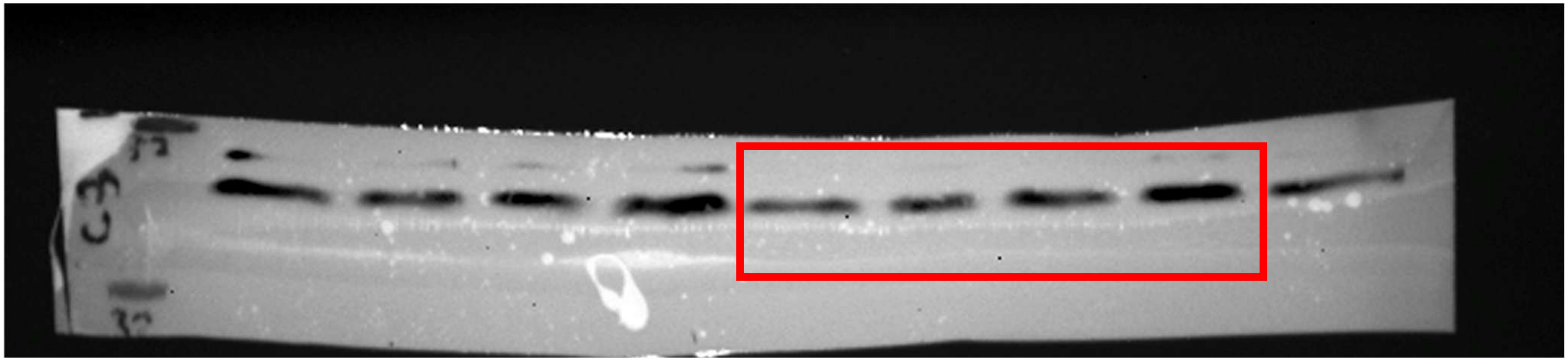

Blot Order

SCR – PDCD4 siRNA – SCR – PDCD4 siRNA
